# The grapevine (*Vitis vinifera* L.) floral transcriptome in Pinot noir variety: identification of tissue-related gene networks and whorl-specific markers in pre- and post-anthesis phases

**DOI:** 10.1038/s41438-021-00635-7

**Published:** 2021-09-01

**Authors:** Alessandro Vannozzi, Fabio Palumbo, Gabriele Magon, Margherita Lucchin, Gianni Barcaccia

**Affiliations:** Department of Agronomy, Food, Natural Resources, Animals and Environment (DAFNAE), University of Padova, Campus of Agripolis, V. le dell’Università 16, 35020 Legnaro, Padova Italy

**Keywords:** RNA sequencing, Transcriptomics

## Abstract

The comprehension of molecular processes underlying the development and progression of flowering in plants is a hot topic, not only because that often the products of interest for human and animal nutrition are linked to the development of fruits or seeds, but also because the processes of gametes formation occurring in sexual organs are at the basis of recombination and genetic variability which constitutes the matter on which evolution acts, whether understood as natural or human driven. In the present study, we used an NGS approach to produce a grapevine flower transcriptome snapshot in different whorls and tissues including calyx, calyptra, filament, anther, stigma, ovary, and embryo in both pre- and post-anthesis phases. Our investigation aimed at identifying hub genes that unequivocally distinguish the different tissues providing insights into the molecular mechanisms that are at the basis of floral whorls and tissue development. To this end we have used different analytical approaches, some now consolidated in transcriptomic studies on plants, such as pairwise comparison and weighted-gene coexpression network analysis, others used mainly in studies on animals or human’s genomics, such as the tau (τ) analysis aimed at isolating highly and absolutely tissue-specific genes. The intersection of data obtained by these analyses allowed us to gradually narrow the field, providing evidence about the molecular mechanisms occurring in those whorls directly involved in reproductive processes, such as anther and stigma, and giving insights into the role of other whorls not directly related to reproduction, such as calyptra and calyx. We believe this work could represent an important genomic resource for functional analyses of grapevine floral organ growth and fruit development shading light on molecular networks underlying grapevine reproductive organ determination.

## Introduction

Grapevine (*Vitis vinifera* L.) is indisputably one of the most popular crops in the world. According to the most updated data of the International Organization of Vine and Wine^1^, vines are spread over an area of more than 7 million hectares and produce annually about 75 million tons of grapes, destined to production of wine, table grapes, juices, and raisins (OIV). Therefore, in a sector that alone is worth billions of dollars, a deep comprehension of each of the thorny phases characterizing berry development is pivotal and several approaches have been applied to elucidate the changes that take place from flowering to ripe berry.

From a molecular point of view, analyses of large gene expression datasets represent a key tool to decipher the biological processes underlying the development of a specific tissue or organ. This would explain the exponential increase in the number of expression atlases developed in recent years. An expression atlas should be meant as a snapshot of the genes expressed in one or more tissues, organs, or even cells at a given phenological stage or throughout a developmental kinetics^2^. According to the main databases dedicated to this purpose—the Bio-Analytic Resource (BAR) for plant biology^3^ and the Expression Atlas platform^2^—expression atlases are available for at least 29 plant species. Among the monocots species used to produce the most recent and complete atlases—in terms of tissues and time point analyzed—stand out *Zea mays*^4^, *Sorghum bicolor*^5^, and *Hordeum vulgare*^6^. Instead, for dicots species, in addition to *Arabidopsis thaliana*^7^ a remarkable effort was done in *Glycine max*^8,9^, *Solanum lycopersicum*^10^*, Solanum tuberosum*^11^, and *Vitis vinifera* L. As regards this latter, several RNA-seq-based experiments have been conducted in the last 10 years. Considering the “time” variable, transcriptional profiles in temporal kinetics are available for berry as a whole (Corvina cv.)^12^, grape skin (Cabernet Sauvignon cv.)^13^, and leaf (Summer Black cv.)^14^. Considering the “cultivar” variable, the grape berry transcriptomes of ten different cultivars were compared to identify cultivar specific-splicing events^15^, while Ghan et al.^16^ applied the same approach in 7 wine grape cultivars to identify the common transcriptional subnetworks underlying the berry skin in the late stages of ripening. Also, the comparison among berry transcriptomic profiles of 5 Italian cultivars, each sampled at 4 progressive phenological phases^17^, led to the identification of common switch genes, that seem to regulate the phase transition during berry ripening. In an exhaustive study, Dal Santo et al.^18^ performed an RNA-seq analysis in two genotypes (Cabernet Sauvignon and Sangiovese) at two developmental stages and cultivated in three different environments over two vintages, in order to elucidate the contribution of genotype, the influence of environment and the effect of their interaction (G×E) on the berry transcriptome. Finally, the most comprehensive atlas so far produced in grapevine is based on 54 samples representing green and woody organs at different developmental phases and it was developed to infer the specific metabolic pathways characterizing each of the samples^19^.

The abundance of transcriptomic data relating to grape berry and its sub-tissues offers an in-depth insight into the molecular processes underlying berry growth and ripening, but it leaves unclarified most of the upstream aspects related to flower development. In fact, except for Fasoli et al.^19^, who considered some of the floral tissues, transcriptional data straddling the anthesis process and related to single grape whorls are lacking. Since the flower gene expression regulation at both temporal and spatial level is the cornerstone for achieving the specification of morphology and physiology of the berry, we attempted to fill this gap by dissecting the transcriptome profiles of six (calyptra, calyx, anther, filament, stigma, and ovary) and four floral tissues (calyx, stigma, ovary, stigma, and embryo) before and after anthesis, respectively. Making use of analytical tools such as the weighted-gene network coexpression analysis (WGCNA), and the analysis tau (τ) and crossing obtained results with already available data, we tried to identify those hub genes and molecular networks that specifically characterize different floral organs or tissues. This analysis made it possible to identify enriched ontological categories in the different tissues under examination and to isolate specific transcription factors expressed exclusively or predominantly in a given whorl. Furthermore, limited to genes expressed exclusively in anther or in stigma, we carried out a de novo *cis-*regulatory elements (CRE) analysis at the promoter level, in order to identify those motifs linked to the tissue-specific expression of selected genes. This original grapevine atlas could represent an important genomic resource for functional analyses of grapevine floral organ growth and fruit development.

## Results and discussion

### Global RNA-seq analysis of grapevine flower tissues

**Fig. 1:**
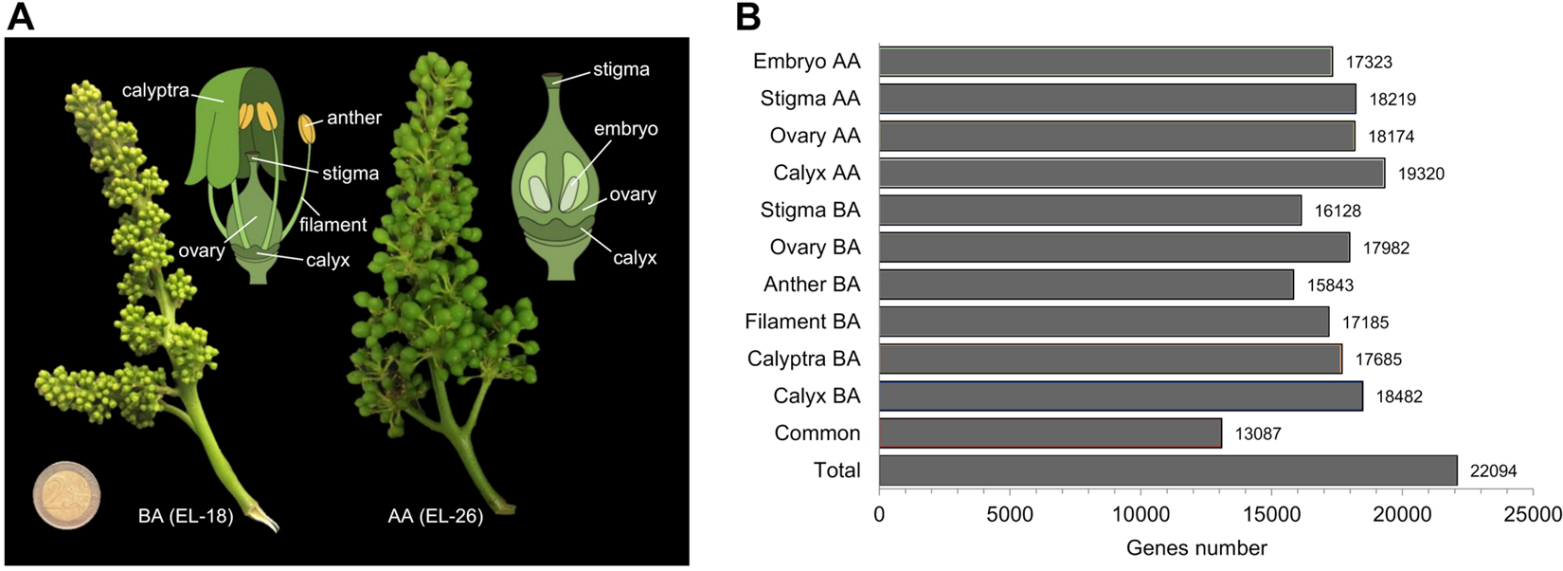
Overview of the *V. vinifera* cv Pinot noir floral samples used for RNA-seq analysis. **A** The pictures show the grapevine inflorescence in pre- and post-anthesis phases, while the schematic illustrations alongside indicate the specific floral tissues sampled for subsequent transcriptional analyses. **B** Number of genes expressed (TPM >1) in each of the 10 tissue/organs. Total: number of genes expressed in at least one organ (22,094). Common: genes expressed in all 10 organs (13,087)

To obtain gene expression profiles for different whorls and tissues of the grapevine flower, RNA sequencing (RNA-seq) data were generated from 10 different tissues collected from flowers of *V. vinifera* cv Pinot noir plants grown in open fields. Six tissue samples were collected from pre-anthesis (“Before Anthesis”—BA) flowers (EL-18), namely calyptra, calyx, filament, anther, ovary, and stigma, and four tissue samples were collected after anthesis (AA; EL-26): calyx, ovary, stigma, and embryo (Fig. 1A). The HiSeq 2500 sequencing run produced a total output of 336 M of 2 × 250 bp reads (on average 12 M reads per sample), while, after filtering steps, ~312 M of reads were retained. Given the low number of reads obtained for Stigma BA and Filament BA, the third replicate from both tissues was excluded from further analyses. The cluster dendrogram analysis based on raw counts (Supplementary Fig. 1) showed a good correlation among the biological replicates of each sample, except for one replicate of Calyx post-anthesis (Calyx AA) grouped with the Embryo samples, which may be due to the high proximity of the tissues which reside very close to each other. The filtered reads deriving from the 28 samples were combined and assembled into a reference catalog, composed by 210,674 transcripts and annotated based on the PN40024 12X v1^20^. After Transcript Per Million (TPM) calculation, 7802 genes were filtered out while 22,094 genes, corresponding to the 73.8% of the total number of genes predicted in the PN40024 12X v1 grapevine reference genome, were retained for further analyses, being expressed in at least one tissue with TPM > 1. Pre-anthesis anther (Anther BA) had the lowest number of expressed genes (15,843), whereas Calyx AA had the highest number (19,320). Finally, 13,087 genes scored a TPM value >1 in all tissues considered (Fig. 1B, Supplementary Table 1).

**Fig. 2:**
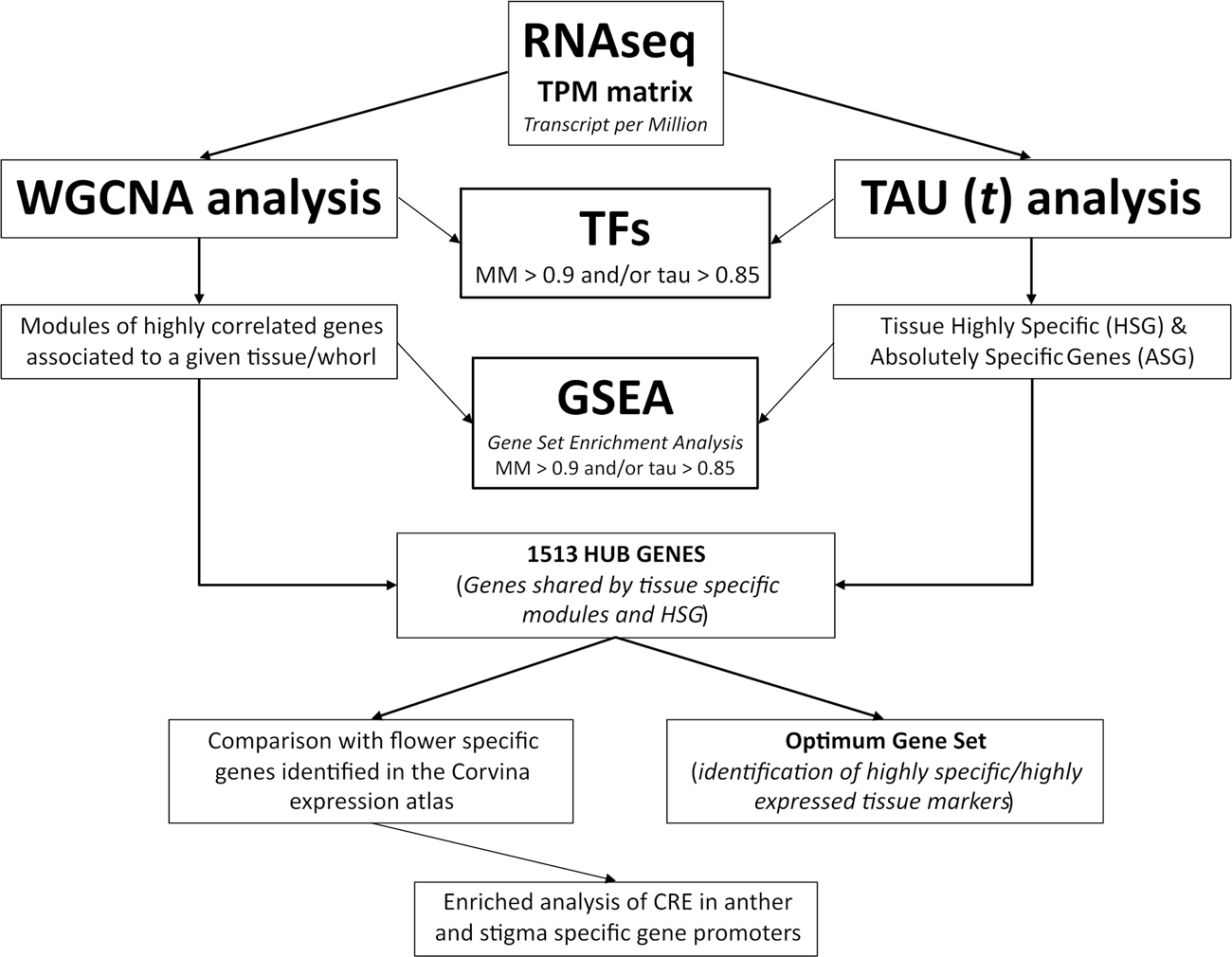
Logical workflow of analyses performed in this study. Schematic illustration of the main analyses performed on TPM normalized data

To isolate tissue-specific genes providing evidence about the molecular mechanisms occurring in different whorls analyzed, we took advantage of different analytical approaches. Some of them are commonly used in plant genomics, such as pairwise comparison and weighted-gene coexpression network analysis (WGCNA), some others have been mainly exploited in animals or human genomics, such as the tau (τ) analysis. For the sake of clarity in Fig. 2, we reported the logical workflow of all analyses performed in this study.

### Weighted-gene coexpression network analysis identified gene modules highly associated with specific grapevine flower whorls/tissues

**Fig. 3:**
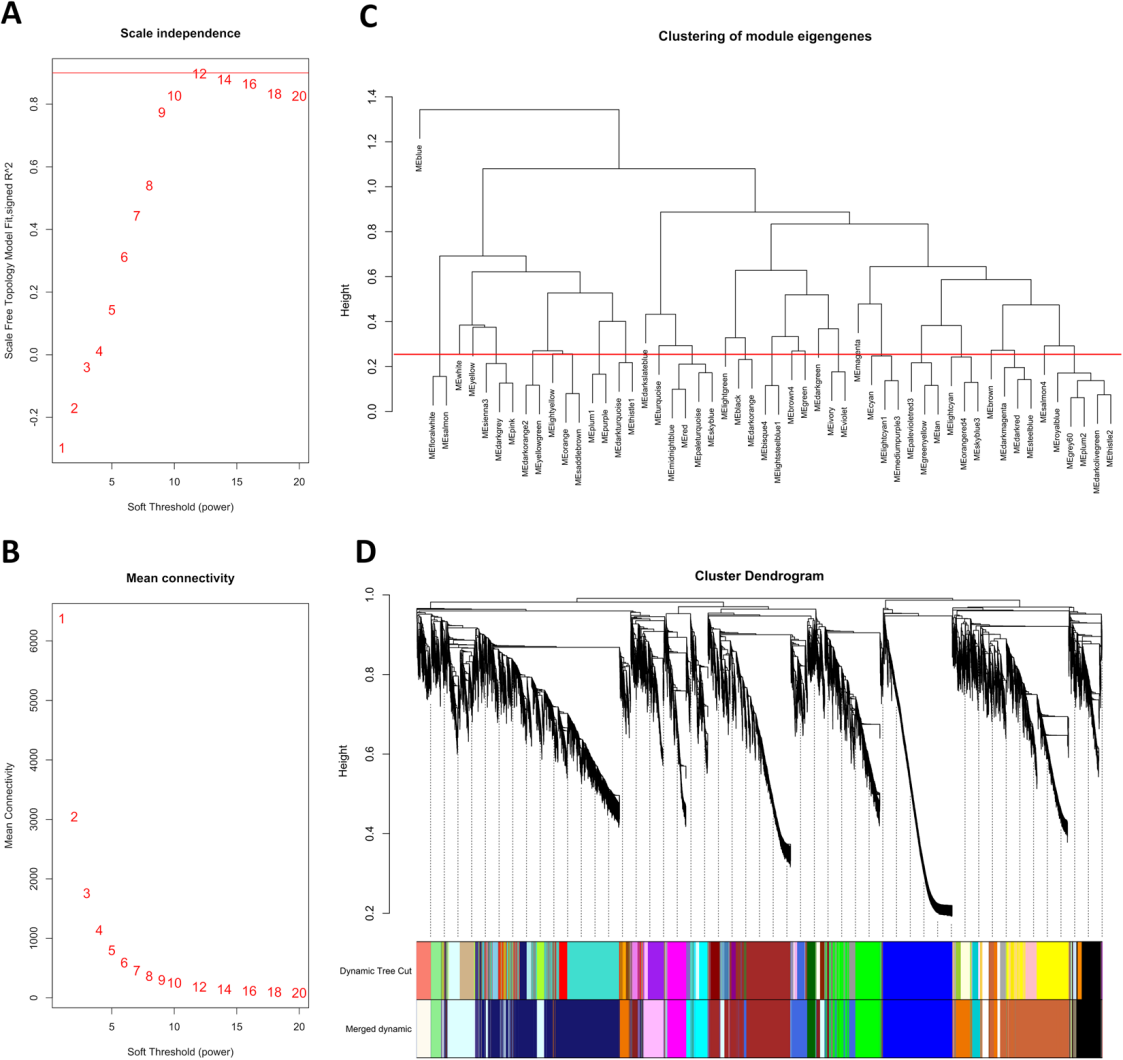
Weighted-gene coexpression network analysis for grapevine RNA-seq data. Analysis of network topology for various soft-thresholding powers showing **A** the scale-free fit index (*y*-axis) as a function of the soft-thresholding power (*x*-axis) and **B** mean connectivity (degree, *y*-axis) as a function of the soft-thresholding power (*x*-axis). **C** Cluster dendrogram of module eigengenes. Branches of the dendrogram group together eigengenes that are positively correlated. The red line is the merging threshold, and groups of eigengenes below the threshold represent modules whose expression profiles should be merged due to their similarity. **D** Hierarchical cluster dendrogram showing co-expressed modules identified by weighted-gene coexpression network analysis for the grapevine flower RNA-seq data. Each leaf on the tree represents a gene. The major tree branches constitute 20 merged modules (based on a threshold of 0.25), labeled with different colors

WGCNA is a systems biology approach aimed at understanding networks of highly correlated genes instead of individual genes, which has been successfully applied in various genomics studies in many plant species including pineapple^21^, strawberry^22^, pear^23^, and grapevine^24^. In this study, coexpression networks were constructed based on pairwise correlations of gene expression trends across all sampled tissues. The 22,094 genes resulting from TPM normalization and filtering, were analyzed in order to identify gene coexpression modules using the R-package WGCNA. Genes showing variance lower than 1 were removed leaving a total number of 19,658 genes. The matrix was raised to a soft-thresholding power *β* = 12 to ensure a scale-free network (Fig. 3A, B). Modules are defined as clusters of highly interconnected genes, such that genes belonging to the same module, highly correlated with each other. For the present analysis, the minimum module size was set to 30, and modules with highly correlated eigengenes (based on a threshold of 0.25) were merged. The eigengene represents the first principal component of a given module and can be considered as a representative of the expression profiles of genes within that module.

**Fig. 4:**
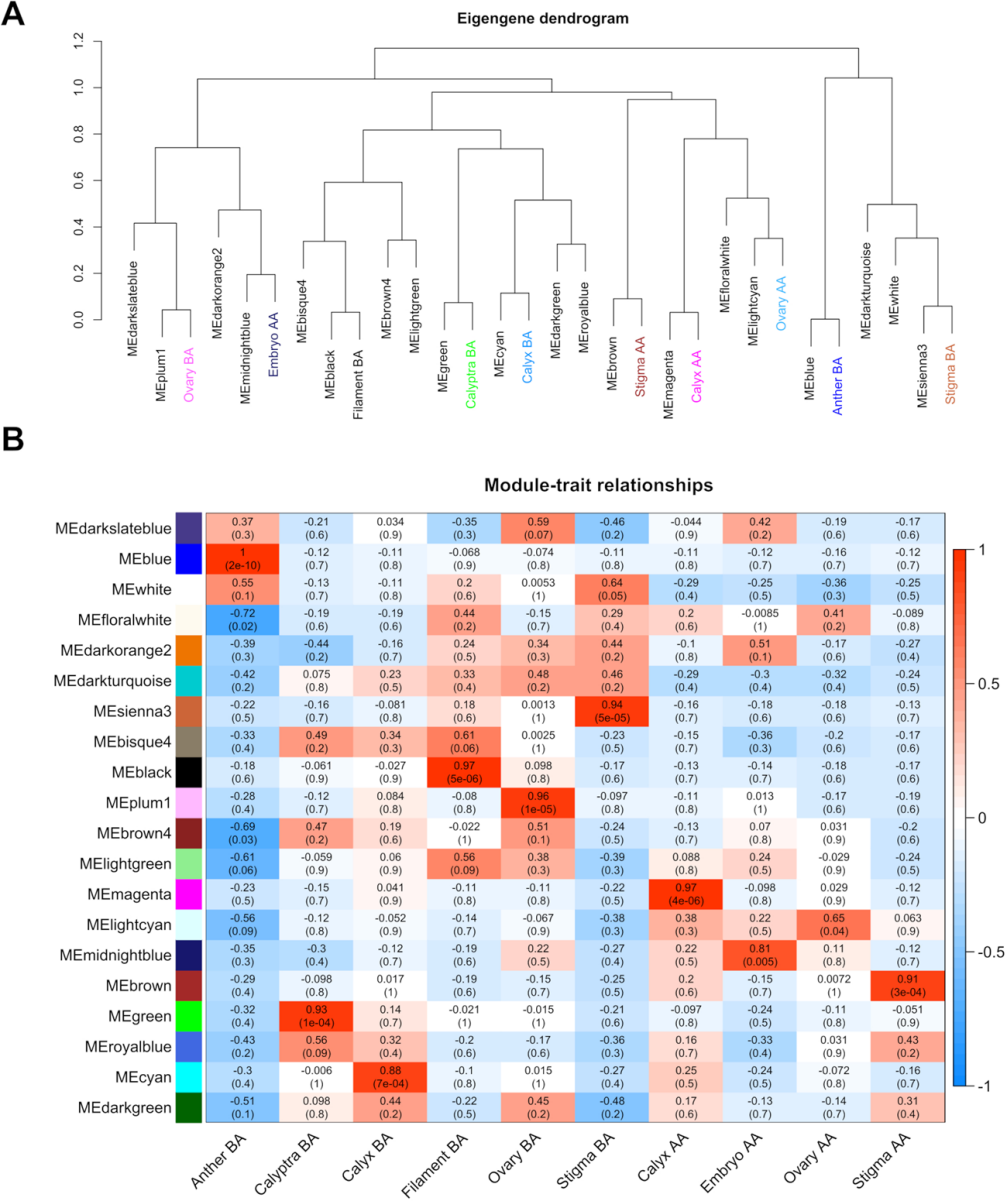
Module-tissue association analysis. Visualization of the eigengene network representing the relationships among the modules and the tissues under study. Panel (**A**) shows a hierarchical clustering dendrogram of the eigengenes in which the dissimilarity of eigengenes E_*I*_, E_*J*_ is given by 1–cor (E_*I*_, E_*J*_). The color of the most correlated module was used to color the name of the organ/tissue. Panel (**B**) heatmap shows the correlation between modules and tissues. Each row corresponds to a module, whereas each column corresponds to a specific tissue. The correlation coefficient between a given module and tissue type is indicated by the color of the cell at the row-column intersection and by text inside cells (*p*-value is also reported). Red and blue indicate positive and negative correlation, respectively

Twenty distinct modules were identified (Fig. 3C). The modules are labeled by color and are shown in a hierarchical clustering dendrogram, in which each tree branch constitutes a module and each leaf in the branch is one gene (Fig. 3D). Next, we performed a correlation analysis between the 20 distinct modules and the 10 tissues/whorls under study (Fig. 4A).

For each organ, at least one highly specific module was identified (*r* > 0.8; correlation *p*-value < 0.01; Fig. 4B), although, in some cases, multiple modules showed significant correlations with the same tissue and/or more tissues showed correlations with the same module. For example, the sienna3, cyan, green, and midnightblue modules were specifically correlated with Stigma BA, Calyx BA, Calyptra BA, and Embryo, respectively. The best correlations between module eigengene (ME) and tissue were between the blue module and Anther BA (*r* = 1, *p* = 2e−10), the black module and Filament BA (*r* = 0.97, *p* = 5e−06) and finally the magenta module and Calyx AA (*r* = 0.97, *p* = 4e−06) (Fig. 4B, Supplementary Table 2). To further investigate the gene constitution of the 10 modules showing the best correlation with tissues under study, two network unique properties such as gene significance (GS) and module membership (MM) were carried out. The module membership (MM) is a measure of the correlation between the expression profile of a given gene with the considered module eigengene. The gene significance (GS) is an additional network parameter, that can be also defined by the minus log of a *p*-value and give an estimation of the biological significance of a gene. The higher the absolute value of GS*i*, the more biologically significant is the *i*-th gene.

**Fig. 5:**
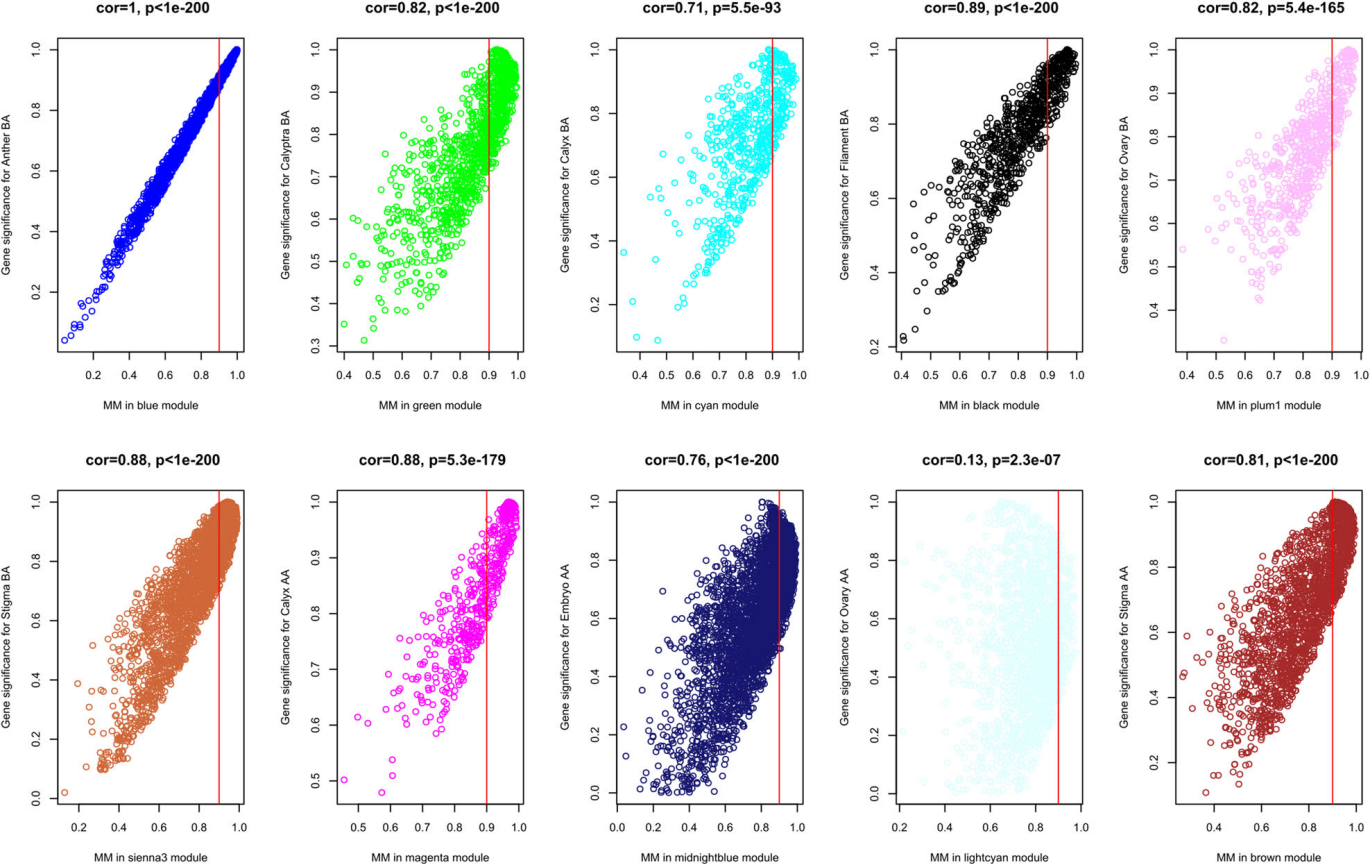
Scatterplots of gene significance (GS) vs. module membership (MM). The plots show the correlation between GS and MM in the ten modules that best correlate with the different tissues analyzed illustrating that gene highly significantly associated with a trait are often also the most important (central) elements of modules associated with the trait. Genes showing a MM >0.9 can be considered as hub genes

**Fig. 6:**
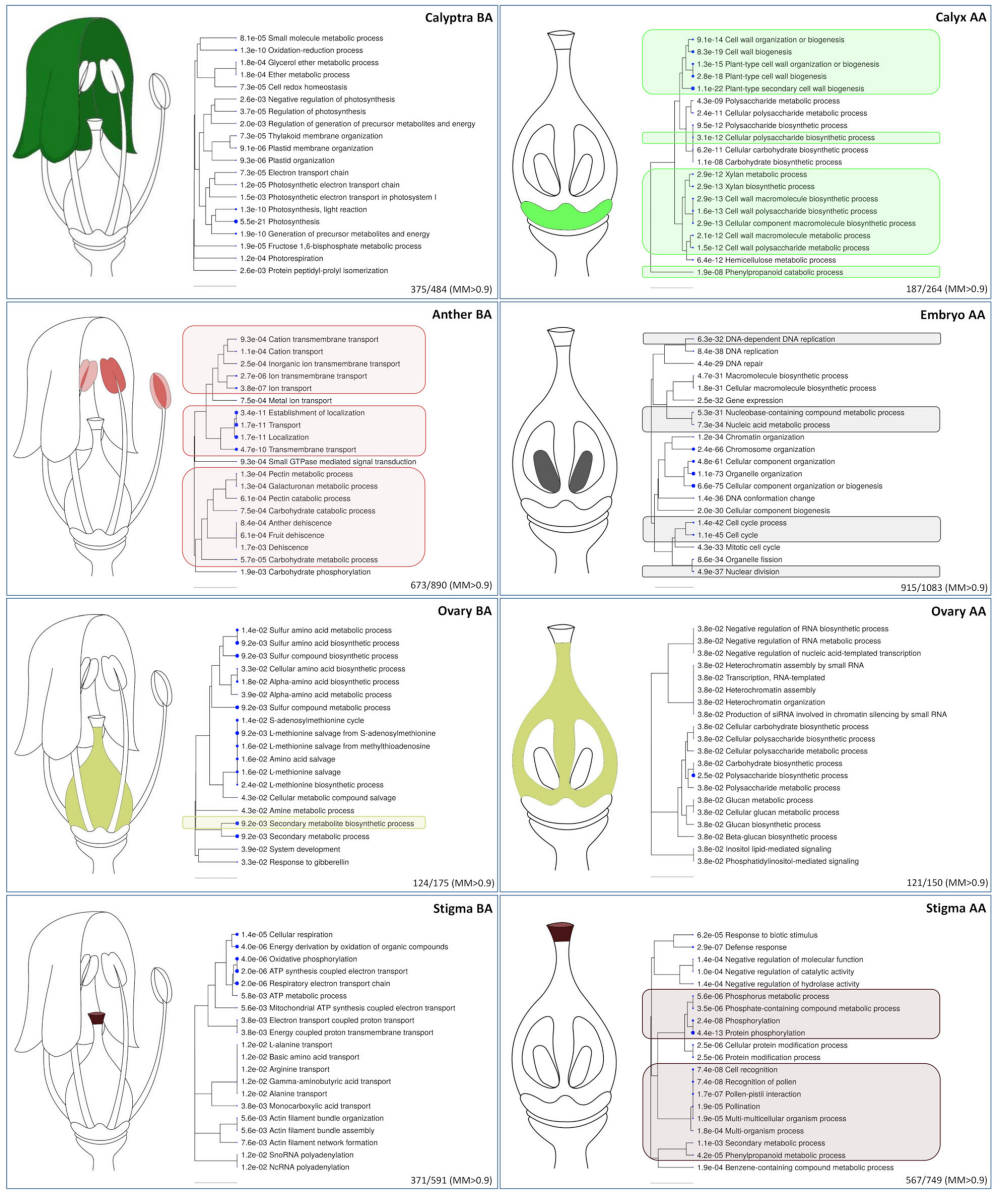
Gene set enrichment analysis. Genes showing a module membership (M) higher than 0.9 were subjected to GSEA and the most relevant biological networks for Anther BA, Calyptra BA, Calyx AA, Stigma BA, Ovary BA, Stigma AA, Ovary AA, and Embryo AA are reported. The hierarchical clustering trees summarize the correlation among significant pathways identified. Pathways with many shared genes are clustered together. Bigger dots indicate more significant *p*-values. GO categories highlighted by colored boxes were detected also in the GSEA on highly specific genes (HSG) obtained by the tau (τ) analysis

Abstractly speaking, if a gene has higher GS and MM, it is more meaningful with the phenotypical trait^25,26^. Thus, a specific module whose MM or GS were significantly connected and associated with the anther tissue may play a more important biological role on anther determination or functionality^26^. Although all 10 modules considered showed extremely significant correlations between GS and MM, the blue (Anther BA), black (Filament BA), and sienna3 (Stigma BA) ones showed the best correlations between MM and GS (Fig. 5). Overall, module blue was observed as the best meaningful module by its strongly positive correlations (*r* = 1, *p* < E−200 in GS vs. MM) indicating its strict involvement in anther specific molecular mechanisms. In order to understand if modules associated with different tissues were enriched in genes belonging to determined ontological categories, we conducted a gene set enrichment analysis (GSEA) on those genes showing a MM >0.9 (Fig. 6), which were considered as WGCNA hub genes.

The analysis allowed to identify the most relevant biological networks for all the tissues except for Filament BA and Calyx AA. This could be due to both the small size of the modules and the absence of specific processes occurring in these whorls. In Calyptra BA we identified a consistent number of biological networks associated with “photosynthesis” (GO:0015979) and “photosynthesis light reaction” (GO:0019684). Within these GO categories, two genes encoding light-harvesting chlorophyll A-binding proteins (*LHCA1* and *LHCA4*) stand out. Their expression resulted sensibly higher in calyptra (1843 and 1153 TPM, respectively; Supplementary Table 3) compared to any other tissue, in accordance with the active role of this green tissue in the photosynthesis process^27^. The most enriched GO categories in Anther BA were “localization” (GO:0051179), “transport” (GO:0006810), and establishment of localization (GO:0051234). Something similar was observed in the Corvina expression atlas, where pollen and stamen were characterized by the strong expression of genes related to transport and cell wall structure^19^. In addition, “anther dehiscence” (GO:0009901), “fruit dehiscence” (GO:0010047), and “dehiscence” (GO:0009900) were the most enriched categories in Anther BA. Five polygalacturonase (PG) encoding genes (Supplementary Table 3) were included in all the categories abovementioned and exhibited the highest expression levels in Anther BA. *VIT_13s0064g00760*, a member of the polygalacturonase GH28 sub-family, showed an impressive transcripts accumulation (3310 TPM) in the male reproductive organ whereas its levels were always very low (from 0 to 17 TPM) in all the other tissues. The expression of PG genes in tapetum, pollen grains, stigmas, and pollinated pistils has been described in various species and implies their role in tapetum degradation, pollen maturation, pollen tube growth, and pollination^28^. In Arabidopsis, suppressing the expression of *QRT3* interferes with microspore separation after the tetrad stage^29^, whereas in Chinese cabbage *BcMF6* silencing determines smaller floral organs and a lower pollen germination rate caused by the disruption of microspore maturation^30^. In the same species, *BcMF2* and *BcMF9* RNA antisense lines showed disturbed development of the pollen wall intine layer and of the pollen tube wall^31,32^ and the downregulation of *BcMF2* caused pollen deformity and balloon-like swelling in the pollen tube tip, together with premature tapetum formation^31^. When a soybean PG is heterologously overexpressed in Arabidopsis, inflorescence mortality is over 50%, and siliques and seeds significantly decrease in number^33^. With regards to post-anthesis tissues, “recognition of pollen” (GO:0048544), “pollen-pistil interaction” (GO:0009875) and “cell recognition” (GO:0008037) were unarguably the most interesting terms enriched in Stigma AA. Most of the genes highly expressed in this tissue (and scarcely or no detected within the other tissues), as expected, were linked to the self-incompatibility locus (S-locus) and mainly represented by kinases. In Stigma AA, of interest was also the detection of 99 genes included in the “protein phosphorylation” (GO:0006468) and “phosphorylation” (GO:0016310) categories, both involved in pollen/stigma interaction processes^34,35^. Calyx AA showed a significant number of genes covering categories such as “plant-type secondary cell wall biogenesis” (GO:0009834) and “cell wall biogenesis” (GO:0042546). Among them, most noteworthy is the almost exclusive expression of three distinct genes (*VIT_06s0004g03050*, *VIT_08s0040g01970*, and *VIT_08s0040g02030*) all belonging to the fasciclin arabinogalactan-proteins (FLA11) family. In this regard, the Arabidopsis orthologous (*AT5G03170*) seems to be pivotal for tensile strength and tensile modulus of elasticity, two features that match with the mechanical sustain role of calyx after fertilization and fruit set. Finally, the midnightblue module, the one that best correlates with Embryo AA, included genes mainly involved in cell ontogenesis such as “cellular component organization or biogenesis” (GO:0071840), “organelle organization” (GO:0006996), and “chromosome organization” (GO:0051276), coherently with the intense embryonal development activity (Supplementary Table 3).

### WGCNA and tau analyses identified whorl-specific transcriptional regulators

**Fig. 7:**
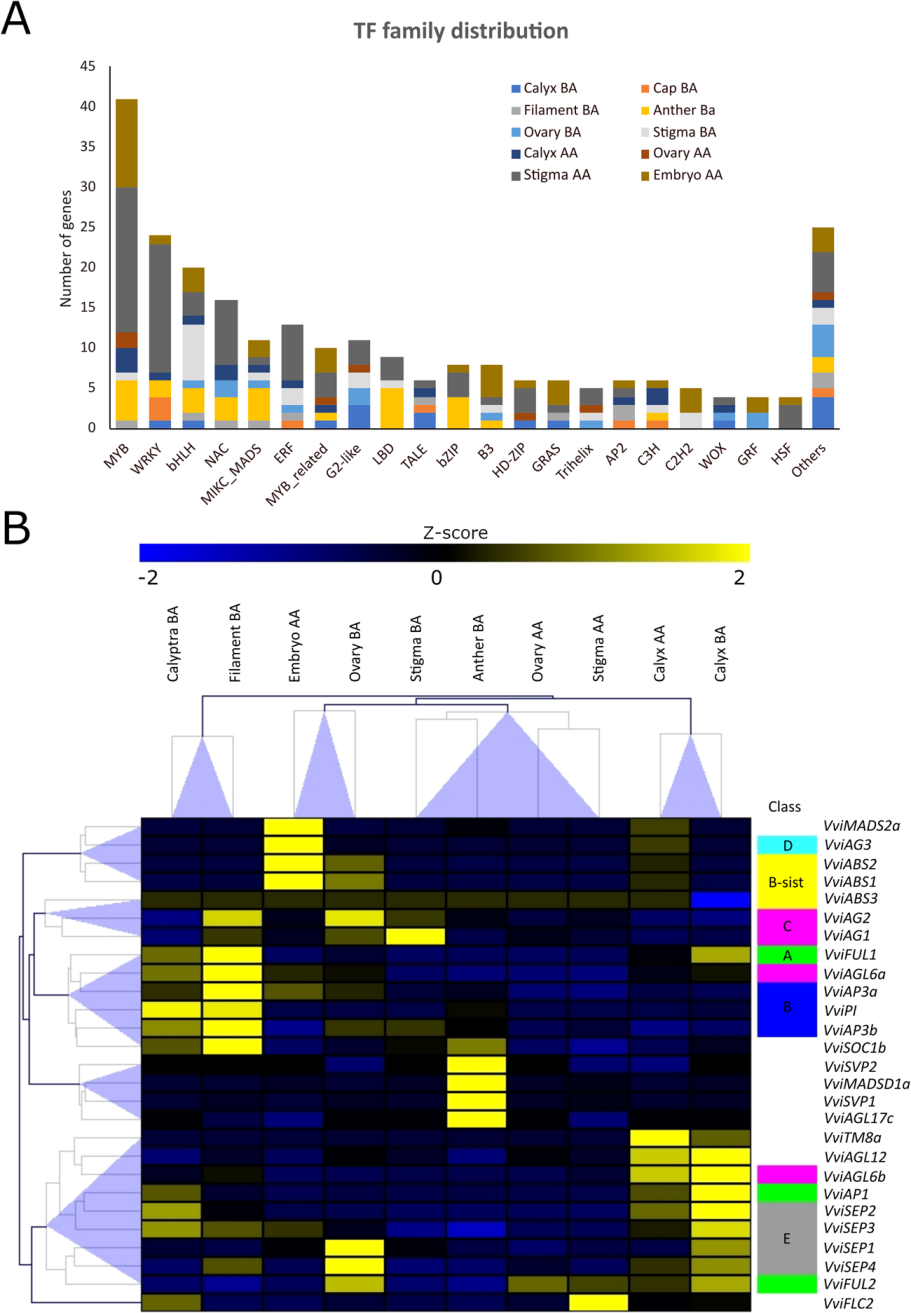
Tissue-specific transcription factors based on WGCNA. **A** Distribution of tissue-specific TF families across the 10 tissues related WGCNA modules. Only TF having a module membership higher than 0.9 was considered. **B** Heatmap showing the behavior of the main homeotic genes described in ref. ^40^ together with those additional MADS box identified by the WGCNA analysis. Data were normalized using the gene/row normalization provided by T-mev software. This approach transforms values using the mean and the standard deviation of the row of the matrix to which the value belongs, using the following formula: Z-score = [(value) – mean(row)]/[standard deviation(row)]. Colored boxes close to gene names indicate the homeotic class of appartenance (for genes that have one attributed to). Hierarchical clustering of both genes and samples grouped genes/samples showing similar behavior

In order to identify transcriptional regulators specifically expressed in different tissues and whorls analyzed in this study, hub genes belonging to the different tissue-specific modules showing MM > 0.9, were screened based on functional annotation reported in the Plant Transcription Factor database (Plant TFDB)^36^. Considering specific genes identified by WGCNA we selected 251 TF genes representing 36 TF families. In absolute terms, the Stigma AA, Embryo AA, and Anther BA were the tissues with the largest number of specific TF-coding genes (83, 40, and 31, respectively). In contrast, Ovary AA and Calyptra BA were the tissues with the lowest number of specific TFs (7). Overall, the MYB-R2R3 (41 genes), WRKY (24), bHLH (20), NAC (16), MICK-MADS (12), and ERF (12) families were the most abundant, although with differences depending on the tissue considered (Fig. 7A; Supplementary Table 4). For example, whether in Stigma AA there was a consistent number of MYB-R2R3 (18) and WRKY (16), in Ovary BA they were lacking, leaving room to genes belonging to minor TF families, such as B3 or G2-like. While in Supplementary Table 4 we listed all tissue-specific TFs identified, in Table 1 we reported the most relevant ones based on module membership (MM > 0.9) and expression. Overall, a rather large number of transcription factors identified appeared to be related to roles in flower development, determination, or identity. Amongst them it is worth mentioning *VviMYB108A* (*VIT_05s0077g00500*)^37^, highly expressed in Anther BA, whose Arabidopsis orthologous is a JA-inducible TF gene with an important role in stamen development and male fertility, being involved in three main aspects: filament elongation, anther dehiscence, and pollen viability^38^. One of the most expressed TFs in stigma BA, the bHLH gene *BEE1*, is orthologous of the BR-responsive gene *AtBEE1* that regulates stigmatic cell development in Arabidopsis^39^. The occurrence of *VIT_17s0000g03580*, another BR-responsive bHLH, in stigma best ranked TFs, raises questions about the involvement of these hormones in the processes of flower development and fertilization.

**Table 1:**
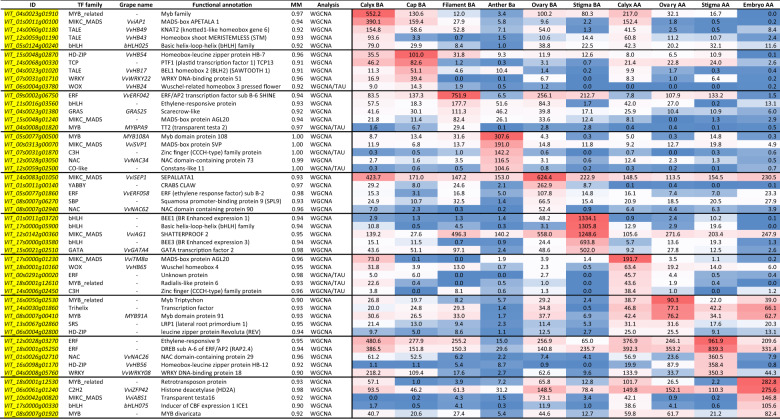
Top-5 tissue-specific TFs identified by WGCNA analysis (MM > 0.9) ordered by descending TPM values

To facilitate understanding, cells are subjected to conditional formatting using a 3-colors scale (blue to white to red), where the lowest values are indicated in blue and the highest in red

Vogler et al. hypothesized that the growth-promoting properties of the reproductive tract of Arabidopsis depend, at least partly, on BR compounds, which are provided by the cells of the reproductive tract to promote pollen germination on the stigmatic papillae, and to boost pollen tube growth for rapid double fertilization^41^. Another TF, *VviAG1* (*VIT_12s0142g00360*), was highly expressed in stigma BA and its orthologous in Arabidopsis, the MADS-box gene *SHATTERPROOF 2*, was demonstrated to be involved in promoting stigma, style, and medial tissue development^42^. Certainly, a gene family of particular interest when it comes to floral identity is represented by floral homeotic genes, which are the basis of the ABCDE model and are well-studied genes involved in flower development^43^. These genes, belonging to the MICK_MADS family, already characterized at the genomic level by Grimplet et al.^44^, have recently been characterized from their transcriptional sub-functionalization by Palumbo et al.^40^, who selected 18 MADS boxes belonging to different classes (A, B, C, D, E) and analyzed their expression in different whorls during Pinot noir flower development. Within the tissue-specific TF obtained in this study, we identified 12 TF belonging to the MICK_MADS box family. Amongst them, only 5 genes fall within those homeotic genes considered by Palumbo et al.^40^, namely *VviAG1* (*VIT_12s0142g00360*), a class C gene listed in the Stigma BA related module (sienna3), *VviSEP1* (*VIT_14s0083g01050*), a class E gene belonging to the Ovary BA module (plum1), *VviAP1* (*VIT_01s0011g00100*), a class A gene belonging to the Calyx BA module (cyan), *VviABS1* and *VviABS2* (*VIT_10s0042g00820* and *VIT_01s0011g01560*), both belonging to the B-sister class and detected in the Embryo module (midnightblue). The absence of the other homeotic genes studied by Palumbo et al.^40^ from those identified in this study is likely due to the approach used to analyze the data. By filtering by module membership, most of those genes expressed simultaneously in more than one tissue, an intrinsic characteristic of some homeotic genes on which the ABCDE model is based, were in fact set aside. Nonetheless, we identified some other MADS-box genes which appear to be expressed in different tissues and which deserve a thought. Amongst these are *VviAGL17c* (*VIT_00s0211g00180*), *VviSVP1* (*VIT_00s0313g00070*), *VviMADSD1a* (*VIT_07s0031g01140*), *VviTM8a* (*VIT_17s0000g01230*), *VviSVP2* (*VIT_18s0001g07460*), *VviFLC2* (*VIT_14s0068g01800*), and *VIT_15s0048g01240*, an AGL20-like gene. Although some of these genes showed limited expression, some others, such as *VviSVP1*, *VviTM8a*, and *VviFLC2* were significantly induced in specific whorls. *VvSP1* belongs to the Anther BA-specific module (blue) and showed a transcript accumulation ~16 times higher with respect to the mean transcript accumulation of all other tissues. *VviTM8a* was the first ranked TF gene for expression in the calyx AA module (magenta), being ~20 times more expressed than in all other tissues. Finally, *VviFLC2* was expressed preferentially in stigma AA (fold change = 9 compared to other tissues). To provide a global view of the behavior of all homeotic genes of interest, the heatmap in Fig. 7B reports all the genes analyzed by Palumbo et al.^40^, together with those that have emerged from the WGCNA in this study.

*VviAP1*, a class A homeotic gene, was highly expressed in Calyx (both BA and AA) and in Calyptra BA, in agreement with what was observed by Palumbo et al.^40^ and with previous observations in other plant species such as Arabidopsis^45^, *Camellia japonica*^46^, and *Medicago trucantula*^47^. *VviFUL1* (*VIT_17s0000g04990*) and *VviFUL2* (*VIT_14s0083g01030*), the two grapevine orthologues of Arabidopsis *FRUITFULL* (*FUL*)^44^, showed distinctive expression patterns. *VviFUL2* was expressed in ovary and calyx before anthesis, whereas *VviFUL1*, was expression was generally much lower, was expressed in Calyx BA, Calyptra BA and, most intriguing, in Filament BA. Class B genes, namely *VviPI*, *VviAP3a*, and *VviAP3b* confirmed their role in petal and stamen identity with the highest transcript accumulation detected in Calyptra BA and stamen tissues (anther and/or filament). Amongst the B-sister genes, *VviABS1* and *VviABS2* perfectly matched what was expected based on Palumbo et al.^40^, being exclusively expressed in Ovary BA and in embryo. For what concerns the class C genes, *VviAG2* and *VviAGL6a* were preferentially expressed in stamen, at the level of filament, whereas *VviAG1* and *VviAGL6b* were expressed in Stigma BA and in Calyx BA and AA, respectively. *VvAG3*, a class D gene, was switched off in all tissue except for embryo, as previously described by Palumbo et al.^40^ and Boss et al.^48^. Finally, amongst the class E genes, *VvSEP1*-*4* transcripts were accumulated preferentially in Calyx BA and AA, with the exclusion of *VviSEP1* which was only detected in pre-anthesis phase. Moreover, a relevant expression in Ovary BA was detected for *VviSEP1* and *VviSEP4*.

### Isolation of whorls/tissue-specific gene markers using the tau (τ) analysis

The WGCNA analysis, based on the identification of clusters of highly correlated genes sharing similar expression patterns across all samples, allowed the determination of groups of genes closely associated with a specific phenotypic character, represented in this study by a determined floral tissue or whorl.

This analysis has proved particularly effective in numerous studies^21–23^, nevertheless, the fact that a particular gene is highly expressed in one tissue compared to others is a relative parameter and in some cases, it may be more useful to identify genes that are exclusively expressed in one organ and not in others: in other words, specific tissue/organ gene markers. For this purpose, we applied an algorithm generally used in transcriptomic studies on animals or humans. Such an algorithm, defined as tau (τ) algorithm^49^, can determine the tissue-specificity level of each predicted gene of a given genome.

**Fig. 8:**
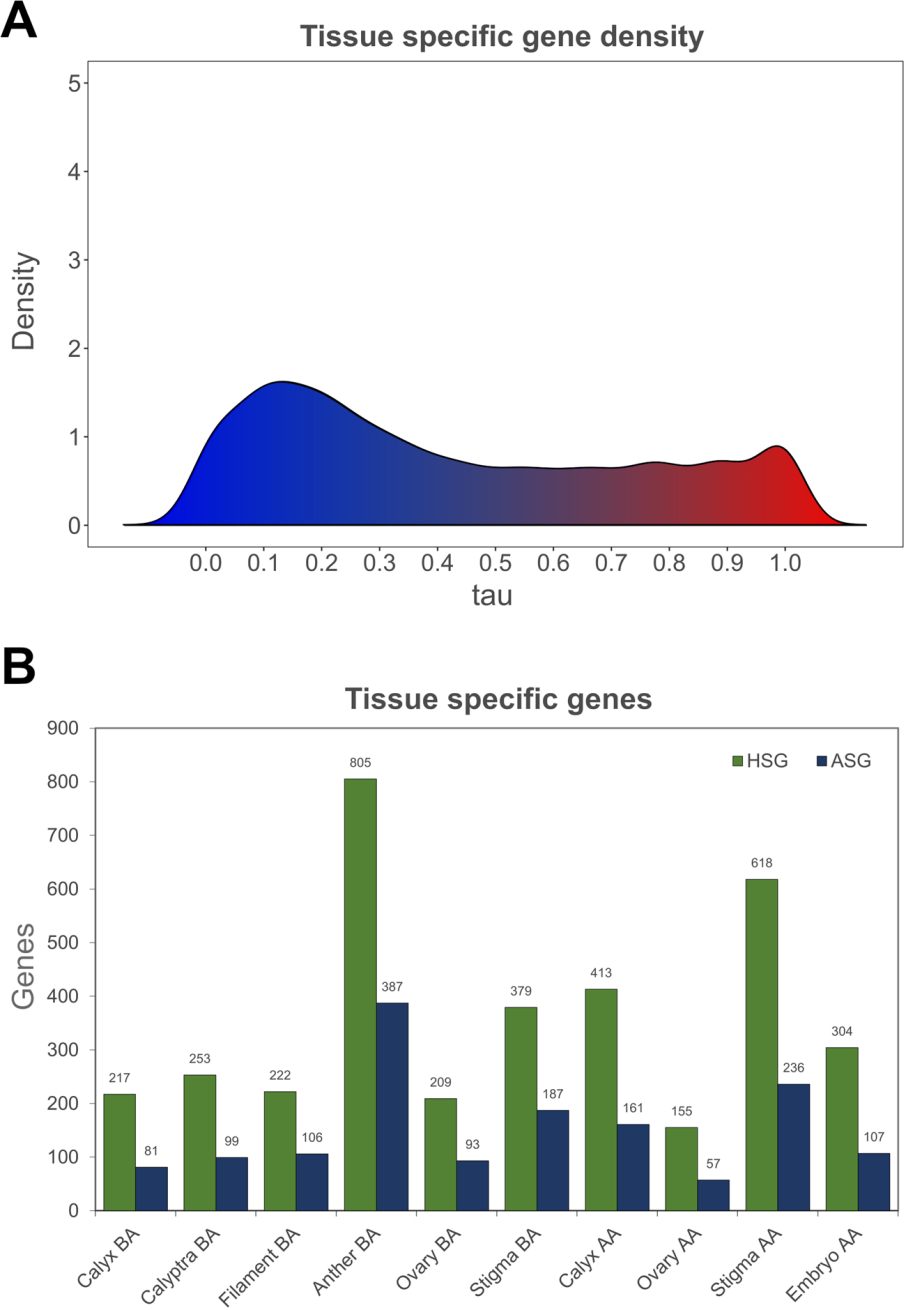
Tissue-specific gene distribution. **A** Distribution of tissue-specificity tau parameter over the 22,094 genes considered (TPM >1). The shape of this plot and density distribution is coherent to what is expected based on ref. ^49^. **B** Bar graph showing the distribution of absolutely specific genes (ASG; tau = 1) and highly specific genes (HSG; tau >0.85) over the ten tissues/organs considered

After the quantile normalization of 22,094 genes (selected because showing TPM values equal or higher than 1 in at least one of the 10 samples) and the creation of BIN profiles, the implementation of the τ algorithm led to the assignment of a value ranging from 0 (constitutively expressed in all or most of the tissues) to 1 (absolutely specific for a given tissue) to each gene. The uneven occurrence of the τ values throughout the gene set is illustrated in Fig. 8A and is coherent with what is expected based on Kryuchkova-Mostacci and Robinson-Rechavi^49^. Overall, 3575 genes proved to be highly specific (HSG, tau >0.85) and, among them, 1514 resulted absolutely specific (ASG, τ = 1). The tau value only defined the “specificity” of a gene, whereas to determine which tissue the gene is specific for, the tau expression fractions (τ_ef_) were calculated.

Anther BA was the tissue displaying the highest number of HSG (805) and ASG (307) while, on the contrary, the lowest HSG and ASG values (155 and 57, respectively) were identified in Ovary AA (Fig. 8B). This observation is intriguing since anther BA represents the tissue that showed the lowest number of expressed genes (TPM> 1; Fig. 1), whereas Ovary AA was one of those tissues with the highest number of expressed genes. Nevertheless, this observation is partially confirmed in the Corvina expression atlas, where, out of 516 genes identified as specific flower, 229, equal to 44%, were specific for stamens and pollen^19^. For each tissue, the list of HSG and ASG is available as Supplementary Table 5. Similar to what was done for the genes obtained through WGCNA, also in this case the highly specific genes (HSG) resulting from the tau analysis were subjected to a GSEA to verify the presence of enriched ontological categories and the possible overlap with those obtained in the WGCNA analysis. As a matter of fact, many enriched terms identified in HSG were common with those highlighted in the WCGNA analysis (Fig. 6). This observation conferred greater robustness to the results obtained and laid the foundations for a subsequent step aimed at further narrowing down the list of key genes of interest.

### Comparison between tau and WGCN analyses and determination of best optimum tissue-specific genes

The comparison between the highly specific genes (HSG) identified by the tau analysis and the tissue-specific modules identified by WGCNA (MM > 0.9) led to the identification of 1513 genes shared by the two approaches. Looking at the specific tissues under study, the percentage of common genes found its maximum in Anther BA (47.4%; Supplementary Table 2; Supplementary Fig. 2). This result is not surprising, considering that the modules isolated by WGCNA contain genes that can show high levels of expression even in those tissues that are not associated with the module itself, whereas highly specific genes (HSG) identified by (τ) algorithm represent genes that are almost exclusively expressed in that specific tissue and not in others. Nevertheless, although the results provided by the two analyses have different biological meanings, we considered those genes shared between the two approaches to be of particular interest, defining them as key hub genes. Ranking of genes by both expression and specificity is useful for anyone working on a single tissue wanting to identify a set of genes that are highly specific to the tissue, that are expressed in high enough quantities (facilitating bench work in the laboratory), and with minimal expression in other tissues (limiting off-target effects). With this aim, we retrieved the quantile normalized expression and tissue-specificity of every key hub gene detected by the WGCNA and tau analyses comparison and we used this information to create a score column. Each gene’s score was between 0 and 2 and was the sum of its tau expression fraction value (τ_ef_) and its 0–1 ranged normalized expression value. For each tissue, we then considered the top 10 ranking genes based on score values, in other words, those genes with both the highest expression and specificity. While the complete list of these genes is available as Supplementary Table 6 and it is graphically represented in Supplementary Fig. 3, in Table 2 we reported the first ranking best optimum specific gene for each different tissue.

**Table 2:**
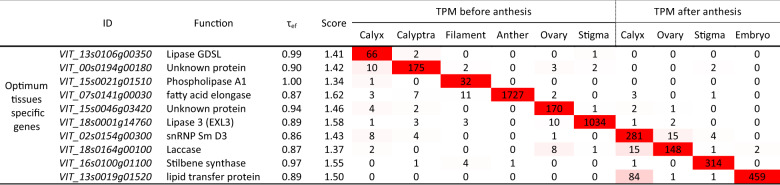
Best optimum gene for each tissue under study based on tau analysis

To facilitate understanding, cells are subjected to conditional formatting using a 2-colors scale (white to red), where the lowest values are indicated in white and the highest in red

Among the most interesting optimum genes in pre-anthesis, the best optimum gene in filament was a *Phospholipase A1* (*VIT_15s0021g01510*; τ_ef_ = 1 and no expression in all the other tissues). Although no information on this gene is available in grapevine, its Arabidopsis orthologous, namely *AT2G44810*, encodes DEFECTIVE ANTHER DEHISCENCE 1 (DAD1), a protein located in the chloroplast^50^ whose phospholipase A1 activity catalyzes the late phase of the jasmonate biosynthetic pathway^51^. *DAD1* expression appears to be restricted to stamen filaments immediately before flower opening and *dad1* mutants show defects in flower opening as well as anther dehiscence and pollen maturation^52^. The massive occurrence of transcript encoding for fatty acid elongase (*VIT_07s0141g00030*) in anther BA (TPM = 1726, compared to an average TPM = 3 in all the others tissues) is in agreement with the grapevine expression atlas published by Fasoli et al.^19^, where this gene was highly expressed in stamen, considering both anther and filament tissues, and in pollen grain. Transcripts of *A. thaliana* orthologous *AT2G26640* encoding a 3-ketoacyl-CoA-synthase 11 (KCS11) are accumulated, above all the other tissues, in stamens and pollen^53^. It is also worth noting that *AT1G68530*, another Arabidopsis orthologous encoding KCS6 another protein belonging to the ketoacyl-CoA-synthase family, is the major condensing enzyme involved in stem wax and pollen coat lipid biosynthesis^54^ and is highly expressed in the tapetum of anthers near maturity^55^. *Kcs6* mutants are male sterile^56^. *VIT_18s0001g14760* transcript was found to be the best optimum gene marker in stigma BA. This observation is consistent with what was observed by Fasoli et al.^19^ in the Corvina expression atlas. The related Arabidopsis orthologous *AT1G75900* encodes a GDSL esterase/lipase EXL3, a protein belonging to the extracellular lipases (EXLs). This class of proteins is abundant in pollen coat, and its combination with lipids can interact with stigma cells, bringing the recognition signal and triggering a mechanical conduit that leads to pollen hydration^57^. It is interesting to note that EXL4, another GDSL esterase/lipase protein, is localized in small granules in the tapetal cells of pollen coat^58^, required for its formation and is involved in male fertility. Mutants show reduced pollen fertility, underdeveloped pollen grain coat as well as impaired water absorption and germination capacities. Amongst the best optimum genes detected in post-anthesis phase, it is noteworthy the presence of a laccase encoded by *VIT_18s0164g00100* in the ovary, as confirmed in the Corvina atlas by Fasoli et al.^19^, where a high expression of this gene was detected in the pericarp and in the pulp of all berry developmental stages, above all in fruit set and post fruit set. The upregulation of *VIT_18s0164g00100* was associated with processes directly involved in berry ripening^24^. Finally, *VIT_13s0019g01520* transcript was highly and specifically accumulated in embryo, again in agreement with the observation of the massive occurrence of this gene mRNA in fruit set and post fruit set seed made by Fasoli et al.^19^. The specific accumulation of a transcript coding for a stilbene synthase, *VvSTS36* (*VIT_16s0100g01100*)^59^, in the stigma AA is curious both for the fact that the induction of this gene and its paralogues had never previously been observed in this tissue, and because generally *STSs* are expressed in response to biotic or abiotic stresses. It is conceivable that in the post-anthesis phase, once fertilization has taken place, the stigma undergoes a senescence process. Several studies reported the accumulation of *STS* transcript and, consequently, of basic and complex stilbenes, in the senescence phase, probably as a response to the oxidative processes and the production of ROS which characterize senescing tissues but also in response to the accumulation of one of the main hormones linked to this process: ethylene, closely linked to the transcriptional activation of these genes. Ultimately, although the discussion of the results obtained only touched on the best optimum genes identified through the tau approach, it seems evident that most of the genes identified found confirmation in the literature. On this basis, through this tool, we believe we have made available a pertinent list of specific tissue/organ genes that may be of interest to the scientific community.

### Integration of the flower and the Corvina expression atlases and Identification of enriched *cis*-regulating elements in flower-specific genes

**Fig. 9:**
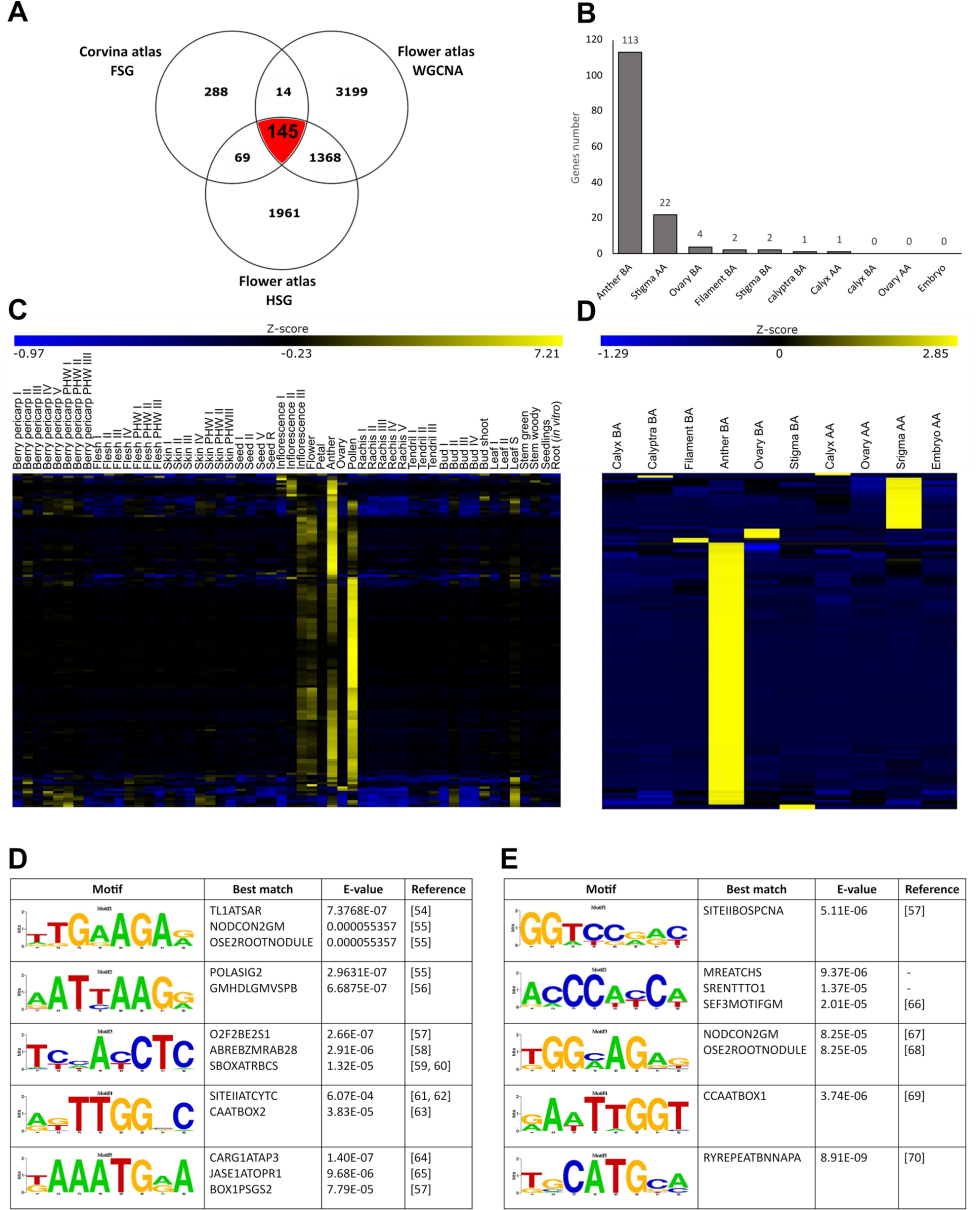
Comparison between flower-specific genes in the Corvina atlas and highly specific genes detected in this study. **A** Venn diagram showing specific or common genes between the Corvina expression atlas^19^ and the highly specific genes detected by tau (HSG) and the WGCNA analyses in this study. One-hundred-forty-five genes were expressed exclusively in flower based on the Corvina atlas and at the same time turned out to be tissue-specific based on the tau and WGCNA analyses. **B** Distribution of the 145 common genes in the different floral tissues based on tissue-specificity; **C** heatmap showing the expression of 145 common genes in the 54 tissues/organs analyzed in the Corvina expression atlas; **D** heatmap showing the expression of the same genes in the flower expression atlas object of this study. For both (**C**, **D**) panels data were normalized using the gene/row normalization provided by T-mev software. This approach transforms values using the mean and the standard deviation of the row of the matrix to which the value belongs, using the following formula: value = [(value) – mean(row)]/[standard deviation(row)]. **E** Top-5 *cis-*regulatory elements (CREs) detected in 113 anther BA-specific genes shared by the three datasets considered. **F** Top-5 *cis-*regulatory elements (CREs) detected in 22 stigma AA specific genes shared by the three datasets considered

Although WGCNA and tau analyses provided lists of genes of interest related to specific floral tissues or expressed exclusively in one whorl rather than another, based on samples considered in this study it was not possible to rule out the fact that these genes are also expressed in other tissues of the plant. In order to further circumscribe the number of genes of interest at the floral level, we did an additional step, excluding those transcripts whose expression was reported also in other tissues based on the *V. vinifera* cv Corvina expression atlas^19^. Based on this analysis, carried out using microarray technology, the number of flower-specific genes (FSG) was estimated 516^19^. We then crossed these data with those resulting from the tau and WGCN analyses, considering all HSG genes (tau >0.85) identified in this study and all those genes belonging to the 10 tissue-specific modules identified in the WGCNA (MM >0.9). Overall, 145 transcripts were found to be common between the three datasets (~28.1% of the specific flower genes identified in the Corvina atlas; Fig. 9A, Supplementary Table 7), representing genes that are expressed only in flower based on the Corvina atlas and specifically expressed in one whorl rather than another based on our data. Two-hundred-eighty-eight genes were expressed only in flower based on the Corvina atlas but did not show specificity for any whorl based on the tau/WGCNA analyses, representing genes that are homogeneously expressed in all flower tissues considered. Of the 145 genes shared by the 3 datasets, the majority was highly specific for Anther BA (113 genes; 78%) and post-anthesis stigma (22 genes; 15%). The remaining ones were HSG for Ovary BA (4 genes), Filament BA (2 genes), Stigma BA (2 genes), Calyptra BA (1 gene), and Calyx AA (1 gene) (Fig. 9B). The heatmaps in Fig. 9 report the behavior of these genes across the 54 tissues considered in the Corvina atlas (Fig. 9C) and in the different floral tissues/whorls considered in this study (Fig. 9D) whereas Supplementary Table 7 provides a list of these key hub genes for each tissue considered.

In order to study the structure of co-expressed genes promoters and to identify genetic determinants of the tissue-specific expression of tissue-specific genes, we conducted a de novo motifs discovery analysis considering the 2 kb sequences upstream the 113 and 22 found to be expressed only in anther and stigma, respectively, based on the WCGNA, tau analysis, and Corvina expression atlas. The analysis was carried out exclusively in these two tissues because they were the only ones having enough genes shared between the WGCNA analysis, the Corvina atlas and the present flower atlas, to allow a reliable de novo motifs discovery. For this purpose, we retrieved the promoter sequences of selected genes previously isolated from the 12x V1 prediction of the PN40024 genome. Using the DECOD software, we screened these sequences for novel enriched motifs with k-mer equal to 8 with respect to 10,000 2 kb promoter sequences randomly retrieved from the grapevine genome. We then used STAMP^60^ to match the motifs discovered against PLACE, a database of motifs found in plant *cis*-acting regulatory DNA elements collected from previously published studies^61^. For both tissues analyzed, 10 motifs enriched in the promoters of tissue-specific genes were identified. The comparison with *cis*-element already deposited in the database via STAMP highlighted the 5 best matches. Among these, we only considered those CREs with a biological meaning.

In Anther BA (Fig. 9E) the first ranked motif based on the score, motif 1 (score = 4.41E−04), was similar to motifs TL1ATSAR (E-value 7.38E−07) and NODCON2GM (E-value 5.54E−05), both enriched in promoter regions of genes involved in reproductive processes. TL1ATSAR was identified in Arabidopsis and is associated with male meiocyte-expressed genes^62^ whereas NODCON2GM is a *cis*-regulatory element of the gene *TM6*, directly involved in stamens petaloidy and flower shape formation In Peonia spp.^63^. Motif 2 (3.26E−04) was associated with POLASIG2 and GMHDLGMVSPB. POLASIG2 is highly related to a large number of flowering genes of Arabidopsis and GMHDLGMVSPB was found to be highly and specifically related to pollen tapetum-expressed genes in rice (*Oryza sativa*)^64^. Something similar was observed for O2F2BE2S1, the best match of Motif 3. The ABREBZMRAB28 motif was found to be enriched in the promoter region of bZIP genes involved in floral development in six strawberry species and it is presumed that they also play important role in the male gametophyte^65^. Moreover, in Arabidopsis, several *AtbZIP* genes were selected for their putative involvement in pollen development^65^. Moving from anther to stigma AA, the other tissue that allowed, given the number of specific tissue genes, to carry out the analysis of the promoters, among the identified motifs whose biological significance has been confirmed by the similarity with already characterized CREs stand out SEF3MOTIFGM, NODCON2GM, and RYREPEATBNNAPA. SEF3MOTIFGM was found in the promoter region of *SEP3*. *SEP3* sequence of *Platanus acerifolia* was heterologously expressed in tobacco and through a GUS assay observed to be expressed in reproductive tissues, among them also stigma^66^. NODCON2GM motif is enriched in the promoter region of the poplar gene *PtaRHE1*, coding for a RING-H2 protein, ectopically expressed in tobacco and observed highly expressed in the stigma through a GUS assay^67^. Finally, RYREPEATBNNAPA in Arabidopsis, was found to be enriched in ABA-related differentially expressed genes which were observed to be highly affected by the overexpression of MINI ZINC FINGER 1 (MIF1), a putative zinc finger protein^68^. For mutants, a lot of defects were observed in floral whorls, including stigma, showing also reduction in fertility^68^.

Ultimately, many of the regions identified through this approach found confirmation in the literature. An interesting investigation would be to evaluate the possible relationships existing between the transcription factors identified through WGCNA analysis (for example *MYB108A*, *VviSVP1*, and *VvNAC34*) and the *cis-*elements identified here. To date, there are several NGS approaches that could shed light on the physical interaction of these *trans-* and *cis*-factors, first the DNA Affinity Purification Protocol (DAPseq)^69^. This could validate the possible interaction between these specific TFs and specific tissue/organ genes and, in association with further RNA-seq analyses, could provide numerous information about the tissue-specific transcriptional networks of the flower, as well as about the cistrome landscape of candidate TFs.

### Isolation of novel housekeeping genes based on the floral and Corvina expression atlases

Looking at constitutive (housekeeping) genes, we identified 662 genes that exhibited a τ value = 0 and TPM values >100 in all tissues. The complete list (in ascending order based on standard deviation of TPM values), is available as Supplementary Table 8, while Supplementary Fig. 4 depicts the main biological networks resulting from the GO enrichment analysis of the 662 housekeeping genes here identified. The GO categories more represented were “positive regulation of translational termination” (GO:0045905), “proteasomal ubiquitin-independent protein catabolic” (GO:0010499), and “assembly of large subunit precursor of pre-ribosome” (GO:1902626) that scored, respectively, fold enrichment values of 34.15, 27.32, and 22.77. It is not surprising that the most represented categories (and the related genes) resulting from the GO enrichment analysis are involved in molecular processes common to all tissues and strictly required for cellular survival (protein synthesis and degradation). A further step was taken to verify whether the 662 constitutive floral genes found in all the tissues here analyzed, were also constitutively expressed in the 54 tissues considered in the Corvina atlas^19^: in this way, we managed to ascertain the qualitative expression of the entire gene list also in the Corvina atlas and to reclassify therefore these loci as “whole plant housekeeping genes”. Due to their crucial role in cellular survival and to their constitutive expression, housekeeping genes or control genes play a decisive role for mRNA levels normalization in qPCR studies. At this aim, several studies attempted the identification of optimal housekeeping genes to be used for specific grapevine tissues^70^, developmental stages^71^, and variable physiological conditions^72,73^. Some of these genes, obtained by cross-checking our RNA-seq data with the Corvina atlas, matched with the findings already available in the scientific literature regarding the ubiquitous expression of genic loci such as *ACTIN 7* (*VIT_04s0044g00580*)^72^, *RIBOSOMAL PROTEIN 60S* (*VIT_18s0001g06410*)^74^, *ELONGATION FACTOR 1-alpha 1* (*VIT_06s0004g03220*)^71^, *GAPDH* (*VIT_17s0000g10430*)^71^, and *AQUAPORIN PIP2B* (*VIT_13s0019g04280*)^71^. On the other side, an exhaustive and novel list of candidate control genes never considered before is presented here. Among the best 10 optimum housekeeping genes (in terms of transcripts abundance and comparable expression levels in all tissues of this study and in the Corvina atlas too; Supplementary Fig. 4 and Supplementary Table 8) stand out four proteasome-related genes, genes involved in exocytosis (*RAB GTPase ARA3*), RNA transcription (*CTV.22*), and protein synthesis (*EIF-3E* and *EIF-4A3*) stands out a member of the Pollen Ole e 1 allergen and extensin (*AtPOE1*) family, initially identified as a group of allergens and recently recognized as developmental regulators in many plant tissues^38^. A further qPCR validation step is needed to evaluate whether these genes could represent a valid alternative to those commonly used in expression analyses.

### Conclusions

Although grapevine is a plant species mainly propagated by agamic way, nowadays, the understanding of the molecular mechanisms that lead to the ontogenetic determination of the different organs within the flower is a topic of great interest. The reasons behind this statement are many: (i) the importance of conventional genetic improvement of varieties as well as rootstocks is increasingly affirming, in contrast to the historical and cultural legacies that see viticulture as an extremely conservative discipline; (ii) flowering represents the first step of the reproductive phase that will lead to the development of the main vine product, the berry; (iii) the structure of the flower and the architecture of the inflorescence influence the organization of the bunch, with many consequences at the production level; (iv) the flower represents the main source of tissues (anthers and filaments) used for in vitro regeneration and, indirectly, for genetic improvement through new breeding techniques. In this study, we generated 30 grapevine RNA-seq datasets for different whorls and tissue of *V. vinifera* cv Pinot noir flowers in pre-anthesis (E–L 18, 8 days before anthesis) and post-anthesis (EL-26, 6 days after anthesis) stages and integrated them with a previously published grapevine cv Corvina expression atlas, which included several flower samples at different development stages^19^. Both WGCNA and tau analysis were used to analyze the RNA-seq data and identify tissue-specific gene modules or marker genes and many of these were identified by both methods. Both analyses have advantages and disadvantages, depending on the objectives of the work and the biological questions to be answered. In this study, which has as its main objective the development of a transcriptomic reference for functional studies on flower-specific genes in grapevine, we tried to combine both approaches, identifying key hub genes which specifically characterize different flower organs before and after anthesis. Although this work is configured as a descriptive study at a wide genome level, the provision of numerous data relating to specific genes for every single tissue or whorls considered represents an important resource for the scientific community of the vine.

## Materials and methods

### Plant material and sample collection

Flower materials (*V. vinifera* L. cv Pinot noir, clone 115, grafted onto Kober 5BB rootstock) were retrieved on May 2018 from a germplasm collection established in 2009 in the experimental farm “Lucio Toniolo” in Legnaro (University of Padova, Padova, Italy; 45°21′5,68″N 11°57′2,71″E). The soil texture was as follows: 46% sand, 24% clay, and 30% loam; pH = 7.9; electric conductivity, 112 μS; and organic carbon, 1.1%. Specifically, in May 14th (E–L 18, 8 days before anthesis) and May 28th (E–L 26, 6 days after anthesis), three inflorescences, each of which collected from an individual plant (1 inflorescence × 3 plants) were sampled and snap-frozen in liquid nitrogen. Each flower was then rapidly dissected in cold conditions into the relative whorls with the aid of a stereoscope and a scalpel. From pre-anthesis flowers, calyptra (or cap), calyx, anther, filament, ovary, and stigma were collected whereas after anthesis—being the cap and the male tissues released—the inflorescence was dissected into calyx, ovary, stigma, and embryo. Considering both stages, ten tissues were isolated, each in three biological replicates (*n* = 30).

### RNA purification, library preparation, and sequencing

For each sample, ~50 mg of tissue were ground in liquid nitrogen, and total RNA was purified using the “Spectrum Plant Total RNA Kit” (Sigma-Aldrich, St. Louis, MO, USA) following the instruction provided by the manufacturer. The integrity of total RNA was checked on 1% (w/v) agarose gel (Life Technologies, Carlsbad, CA, USA) stained with 1 x SYBR Safe DNA Gel Stain (Life Technologies) while the quality (in terms of 260/280 and 260/280 ratios) and the quantity were spectrophotometrically evaluated using NanoDrop-1000 (Thermo Scientific, Wilmington, MA, USA). RNA was stored at −80 °C until use.

cDNA libraries construction and sequencing were performed as described by Chitarrini et al.^75^. Briefly, 1 µg of total RNA was used to construct stranded mRNA-seq libraries (KAPA Stranded mRNA-Seq Kit, Kapa Biosystems, Woburn, MA, USA), that were later barcoded using the KAPA Dual-Indexed Adapter Kit (Kapa Biosystems). Libraries were then quantified (KAPA Library Quantification Kit, Kapa Biosystems) using a LightCycler 480 (Roche, Mannheim, Germany), checked in terms of correct size (250–280 bp) with a Tapestation 2200 (Agilent Technologies, Santa Clara, CA, USA) and High Sensitivity D1000 ScreenTape assay (Agilent Technologies) and, finally, multiplexed. Sequencing was performed on an Illumina HiSeq 2500 platform (Rapid Run Mode, Illumina, Inc., San Diego, CA, USA) to generate 2 × 250 bp reads. All raw reads were deposited in the NCBI SRA database with accession numbers SRR14777742–SRR14777769.

### RNA-seq analysis

FastQC software v.0.11.9^76^ was used to summarize analysis results and to verify the overall quality of the sequencing output while fastp v.0.36^77^ was used to trim the Illumina adapters, merge the reads and filter the sequences based on phred quality score (removed if Q<30). Trinity software v2.8.5^78^ was used in de novo mode to assemble raw reads deriving from the 28 samples (two samples, one from stigma and one from filament were excluded) into a single reference catalog as demanded by Salmon^79^ for quantifying transcript abundance from RNA-seq reads. Trinity was run with default parameter by setting the minimum contig length to 200 and k-mer value at 25. The resulting catalog was then annotated based on the PN40024 12X v1 grapevine reference genome assembly (29,971 genes^80^) and using the BLASTn algorithm^81^. Since the average number of raw reads produced per sample (~12 million) did not reach the minimum threshold required to estimate possible alternative transcripts (i.e., 30–60 million per samples^82^), all the putative isoforms (e.g., i1, i2, i3) produced by Trinity and therefore deriving from the same gene locus (e.g., VIT_18s0166g00210), were annotated under the same transcript name (e.g., VIT_18s0166g00210.01). To quasi-map and quantify RNA-seq reads with Salmon software v.0.14.1^79^, we built an index based on the newly assembled catalog of flower transcripts. The ‘decoys’ option was used to build a *decoy-aware* index by employing the entire genome as the decoy sequence. The RNA-seq reads of each sample were then quantified and their abundance in terms of transcripts per million (TPM) was calculated. As recommended when using the *decoy-aware* index, we used the ‘validateMappings’ option to mitigate potential spurious mapping of reads arising from unannotated genomic loci sequence-similar to annotated transcriptomic loci.

### Weighted-gene correlation-network analysis (WGCNA)

In order to identify clusters (modules) of highly correlated genes attributable to a specific tissue, coexpression networks were constructed using the WGCNA 1.70–3 (https://horvath.genetics.ucla.edu/html/CoexpressionNetwork/Rpackages/WGCNA/) package^83^ in R-studio Version 1.3.1093, R version 4.0.3^84^. The analysis was performed on 19,658 genes showing a mean TPM equal to or greater than 1 in at least one tissue and variance higher than 1, while the remaining 10,230 genes were filtered out. Parameters used in the analysis were set as follows: weighted network, signed; hierarchical clustering tree, Dynamic Hybrid Tree Cut algorithm; power = 12; minModuleSize = 30. As a first step in the analysis, a matrix of pairwise correlations between all genes across the 10 tissues was built. Then, the matrix was raised to a given soft-thresholding power based on the criterion of approximate scale-free topology and pickSoftThreshold function (*R*^2^ > 0.9) to obtain an adjacency matrix. In order to identify modules of co-expressed genes, the topological overlap-based dissimilarity was constructed^85,86^ and used as input to perform the average linkage hierarchical clustering. Modules with highly correlated eigengenes were then merged (mergeCutHeight = 0.25). The association between merged modules and tissues/organs was tested calculating each module eigengene, defined as the first principal component of a PCA on the gene expression of all genes within the module. For each gene, total and intramodular connectivity (function softConnectivity), kME (for modular membership, also known as eigengene-based connectivity), and kME-*P* value were calculated, resulting in 20 tissue-specific modules. Genes belonging to each module were subjected to a GO enrichment analysis using the online tool ShinyGO^87^.

### Identification of tissue-specific genes and genes constitutively expressed in all floral tissues

The tissue-specificity level of each gene was calculated according to the tau (τ) algorithm^88^, which was demonstrated to be the best performing method to measure expression specificity in a benchmark study by Kryuchkova-Mostacci and Robinson-Rechavi^49^. Tau, whose values vary from 0 (broadly expressed) to 1 (tissue-specific), was calculated using the *tispec* R-package (https://rdrr.io/github/roonysgalbi/tispec). Data were first normalized removing all genes whose expression was <1 TPM in any tissue and then, in order to make cross-tissue comparisons possible, a quantile normalization on the entire dataset was accomplished. Thereafter, for each tissue, a BIN value ranging from 0 (lowest expression) to 10 (highest expression) was attributed to each gene. The specificity of each gene (considering all tissues) was calculated implementing the τ algorithm:

$$\uptau = \frac{{\mathop {\sum }\nolimits_{i = 1}^N (1 - x_i)}}{{N - 1}}$$

where *N* is the number of tissues and *x*_*i*_ is the expression value normalized by the highest expression.

Absolutely specific genes (ASGs) were defined as genes expressed in a single tissue only and indicated by a τ value of 1; highly specific genes (HSGs) were genes with relatively highly enriched expression in a few tissues and defined by a τ value of at least 0.85. Finally, genes were considered constitutively expressed in all floral tissues if τ value was <0.2. The plotDensity function was then used to plot the tau value of every gene and visualize which tau values occur most often. Finally, for each tissue, the specificity of each gene was calculated as τ expression fraction (τ_ef_):

$${\uptau}_{{{{\mathrm{ef}}}}} = {\uptau}\frac{{qn}}{{{\mathrm{max}}}}$$

where *qn* is the quantile normalized expression and max is the highest quantile normalized expression. The function getTissue was used to retrieve the quantile normalized expression and tissue-specificity of every gene in each tissue and to create a score value between 0 and 2. This value represents the sum of its τ_ef_ value and its 0–1 ranged normalized expression value. Ranking of genes by both expression and specificity was used to identify a set of 10 optimal genes that were highly specific for a given tissue, highly expressed, and with minimal expression in other tissues. The online tool VENNY 2.1 (https://bioinfogp.cnb.csic.es/tools/venny/) was used to highlight any possible overlapping among HSGs (resulting from the τ_ef_ analysis) of a given tissue and genes belonging to the cluster (resulting from the WGCNA analysis) significantly most associated with the tissue. Finally, a list of constitutive genes was drawn up retaining those loci that—in all tissues—scored a τ_ef_ value =0 and a TPM value >100.

### Identification of *cis*- and *trans*-regulating factors in genes of interest

To identify genes coding for transcription factors, gene IDs identified by WGCNA and tau analysis were screened against the Plant TFDB^36^. To identify *cis*-regulatory element (CRE), the promoter sequences (2 kb) of the specific genes selected for anther BA and stigma AA were retrieved from the 12x V1 annotation of PN40024^20^. The de novo identification of motifs enriched in the promoters of these genes was carried out using the DECOD software^89^, using as a background a collection of 10,000 promoter sequences obtained randomly from the grape genome. The analysis was performed using k-mers of 8 nucleotides (default parameter), a maximum of 10 motifs identified and 50 iterations. Once the enriched motifs were identified, they were submitted to the online tool STAMP^60^, in order to identify any CRE already characterized in previous studies.

## Supplementary information

Supplementary Figure 1

Supplementary Figure 2

Supplementary Figure 4

Supplementary Figure 3

Supplementary Table 1

Supplementary Table 2

Supplementary Table 3

Supplementary Table 4

Supplementary Table 5

Supplementary Table 6

Supplementary Table 7

Supplementary Table 8

## Data Availability

Raw Illumina sequence data were deposited in the National Center for Biotechnology Information (NCBI) and can be accessed in the sequence read archive (SRA) database (https://www.ncbi.nlm.nih.gov/sra). The accession number is PRJNA736298 and includes 28 accession items (SRR14777742–SRR14777769). All data generated or analyzed during this study are included in this published article and its supplementary information files.
