# Supplementary figures and images for: The grapevine (Vitis vinifera L.) floral transcriptome in Pinot noir variety: identification of tissue-related gene networks and whorl-specific markers in pre- and post-anthesis phases
Source: Hortic Res. 2021 Sep 1;8:200. doi: 10.1038/s41438-021-00635-7 (PMC8408131; doi:10.1038/s41438-021-00635-7)

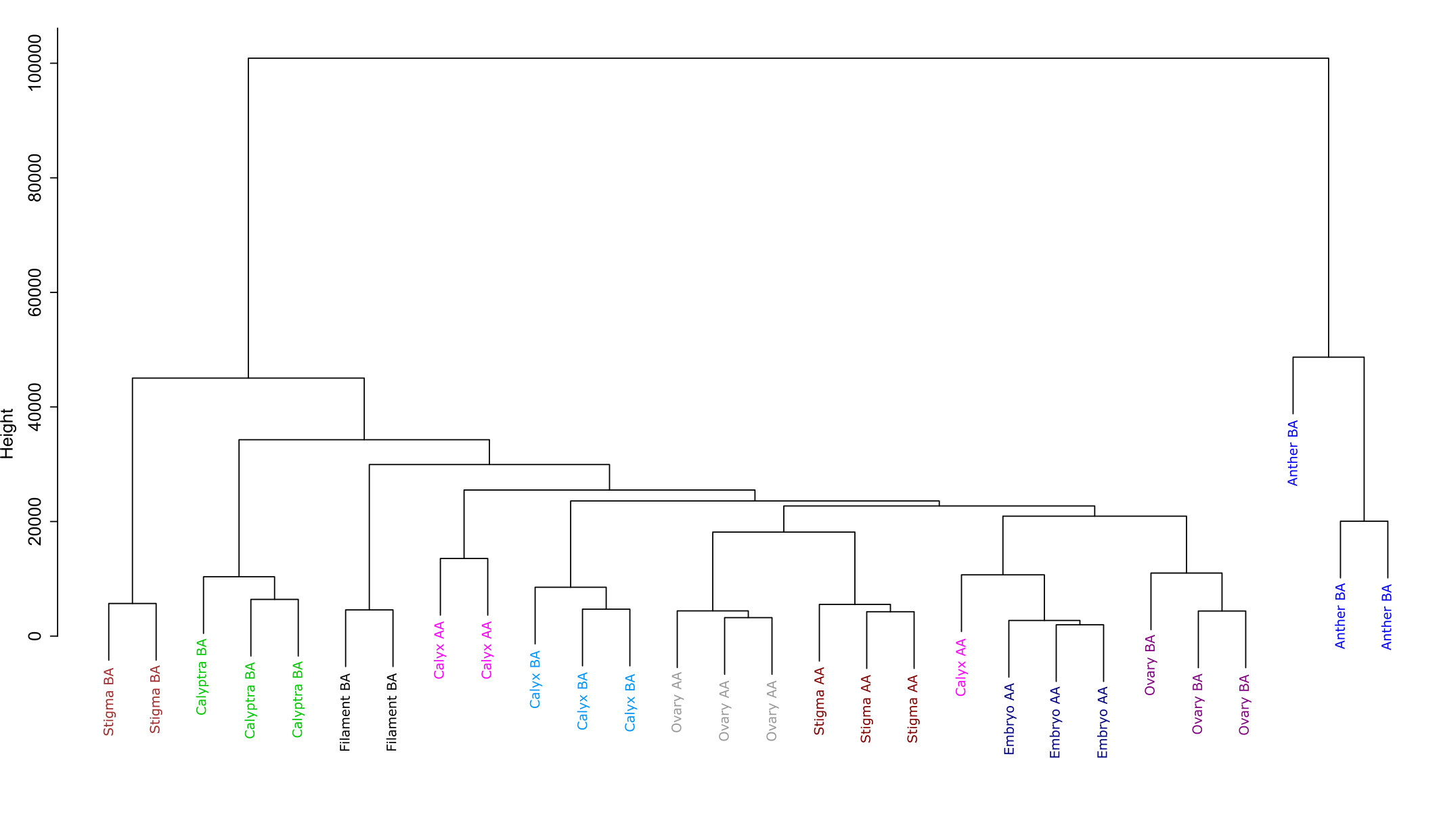

Supplement: Supplementary file 1 — Supplementary Figure 1 [file 41438_2021_635_MOESM1_ESM.jpg]

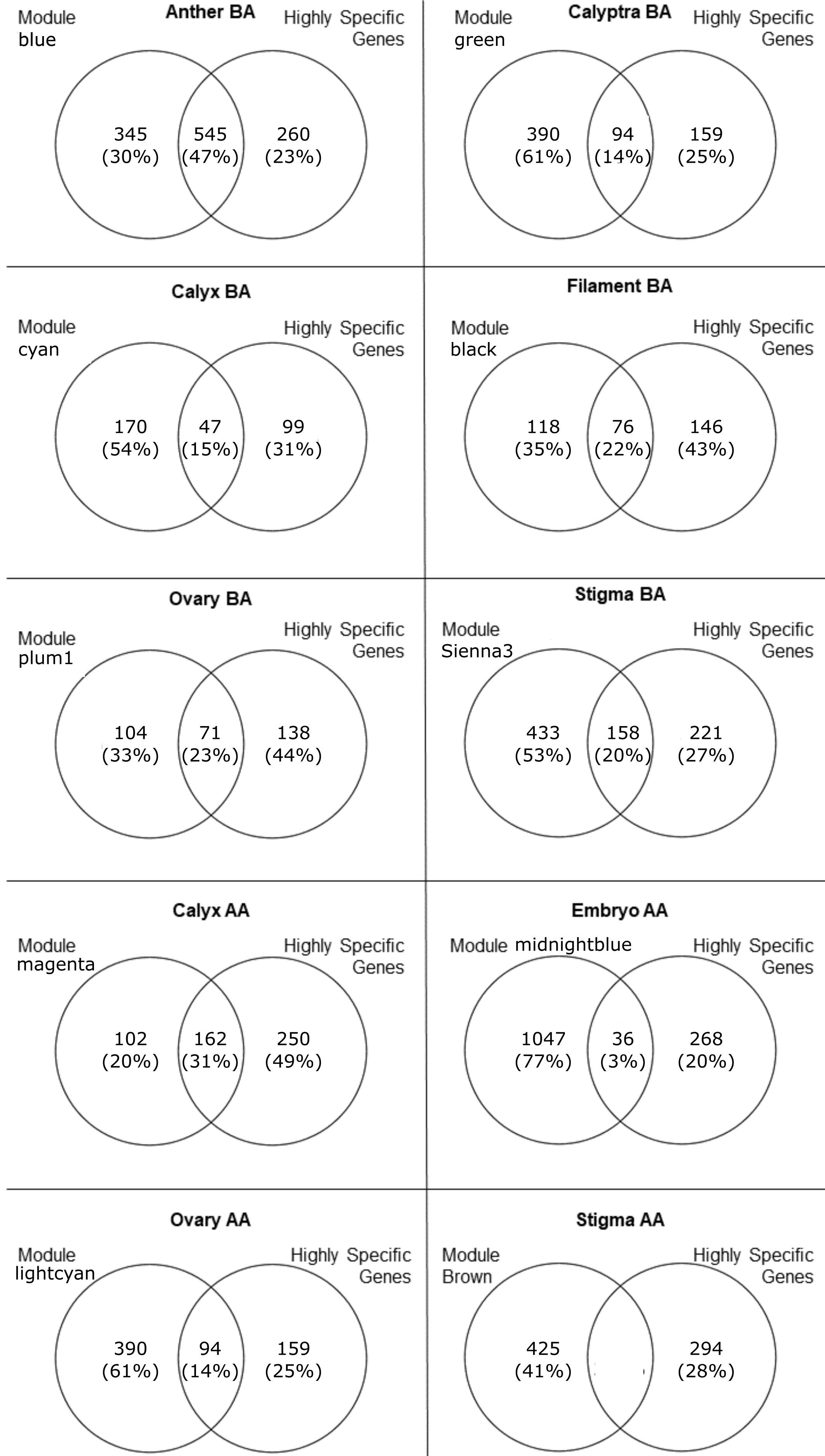

Supplement: Supplementary file 2 — Supplementary Figure 2 [file 41438_2021_635_MOESM2_ESM.jpg]

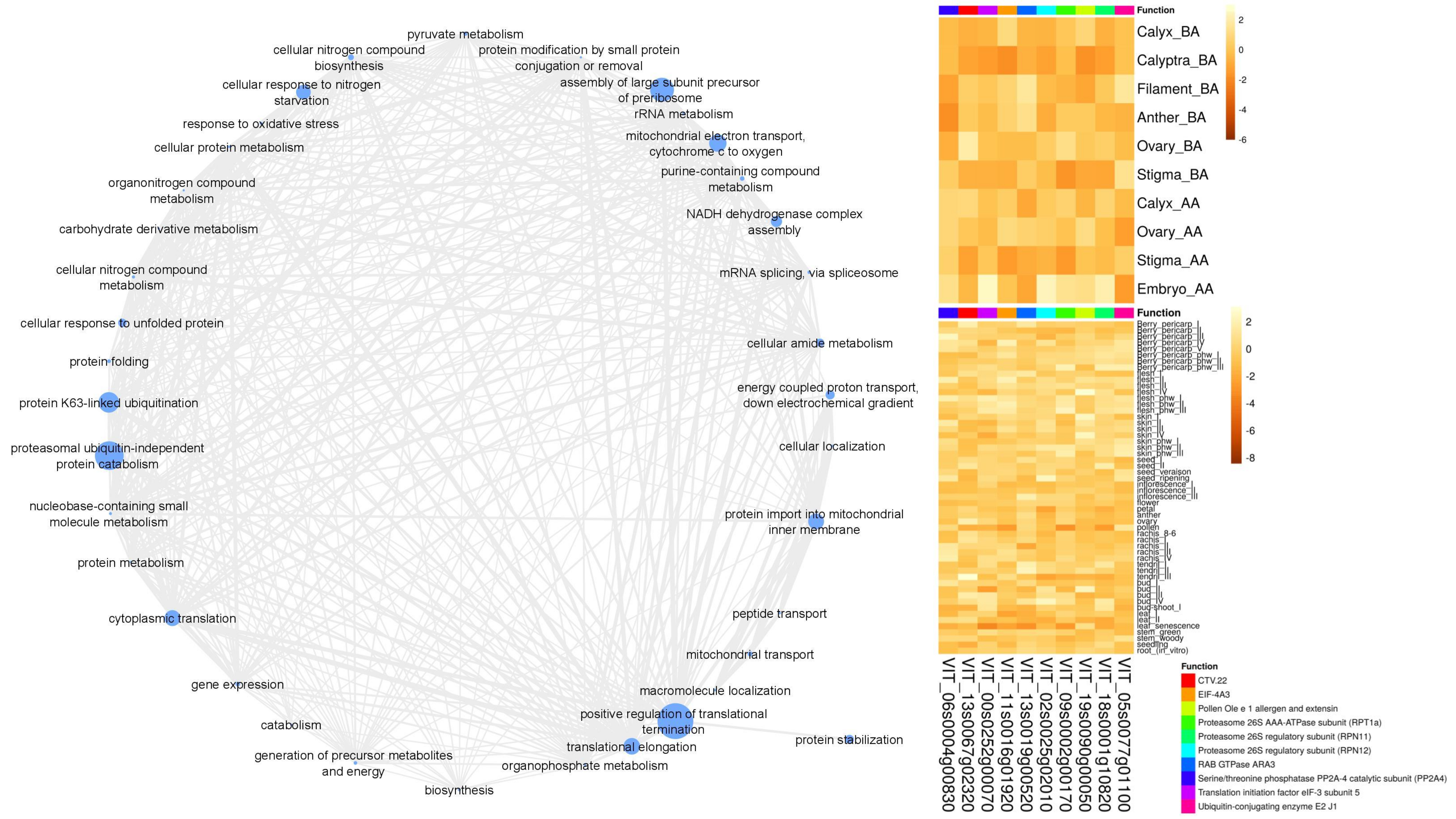

Supplement: Supplementary file 3 — Supplementary Figure 4 [file 41438_2021_635_MOESM3_ESM.jpg]
