# Supplementary material for: The grapevine (Vitis vinifera L.) floral transcriptome in Pinot noir variety: identification of tissue-related gene networks and whorl-specific markers in pre- and post-anthesis phases
Source: Hortic Res. 2021 Sep 1;8:200. doi: 10.1038/s41438-021-00635-7 (PMC8408131; doi:10.1038/s41438-021-00635-7)

Top 10 Gene optimum set (tau expression fraction)

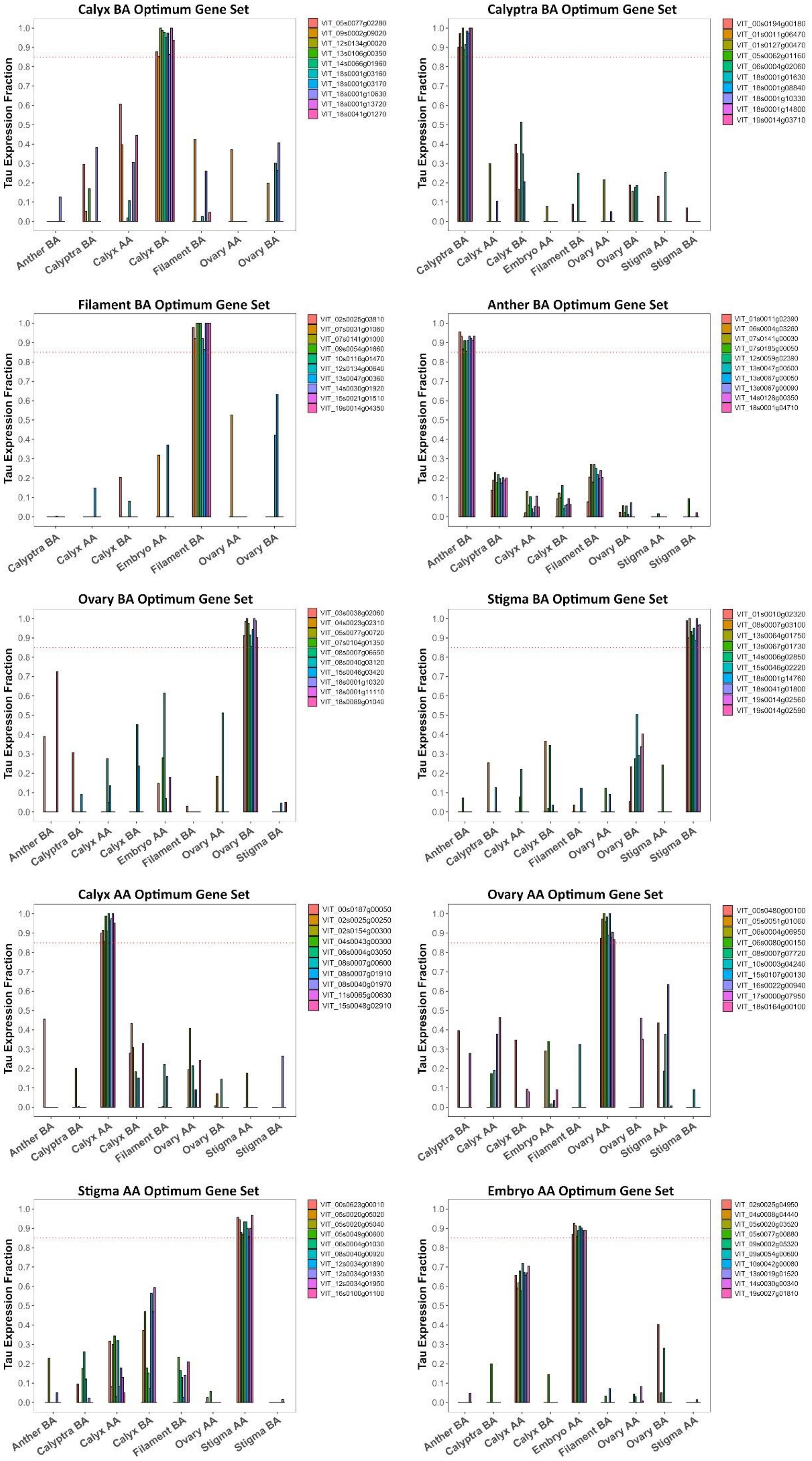

Top 10 Gene optimum set (transcript per Million)

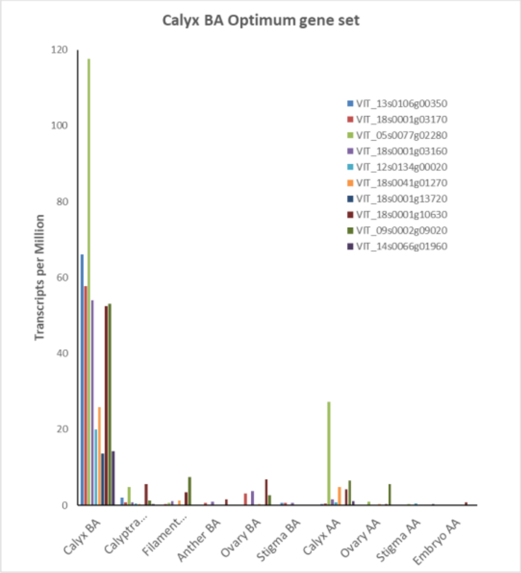

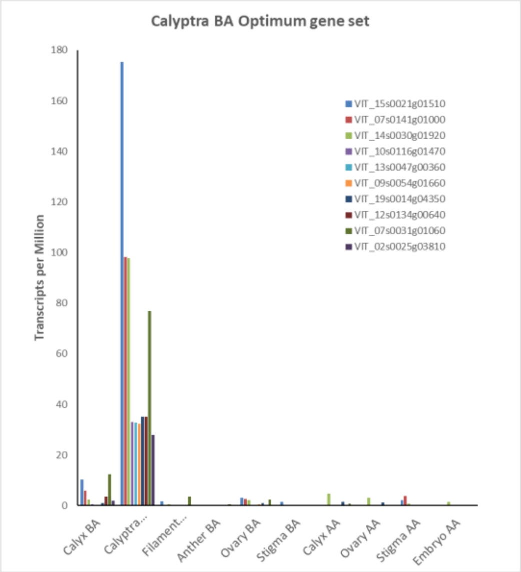

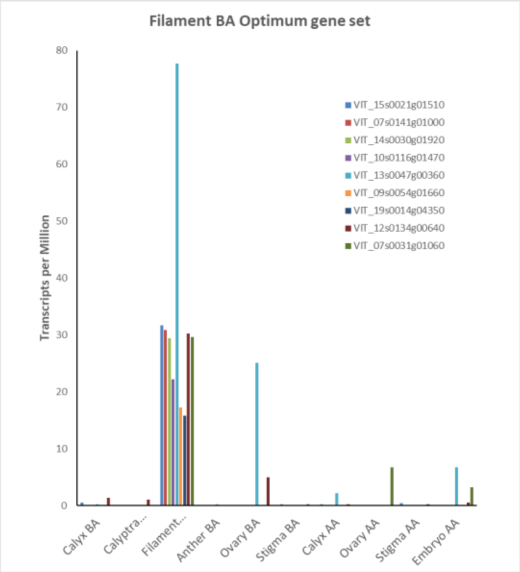

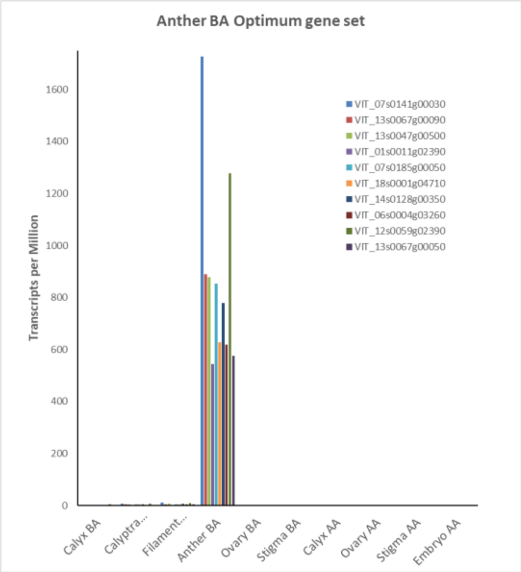

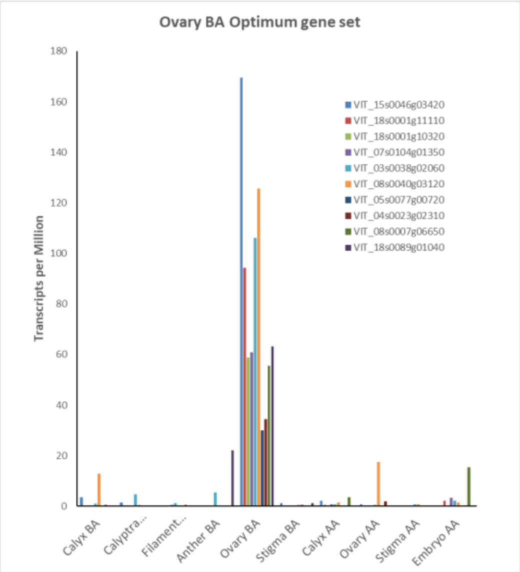

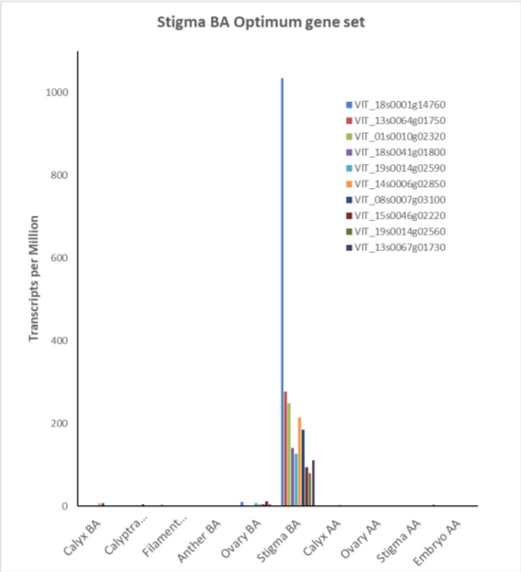

Top 10 Gene optimum set (transcript per Million)

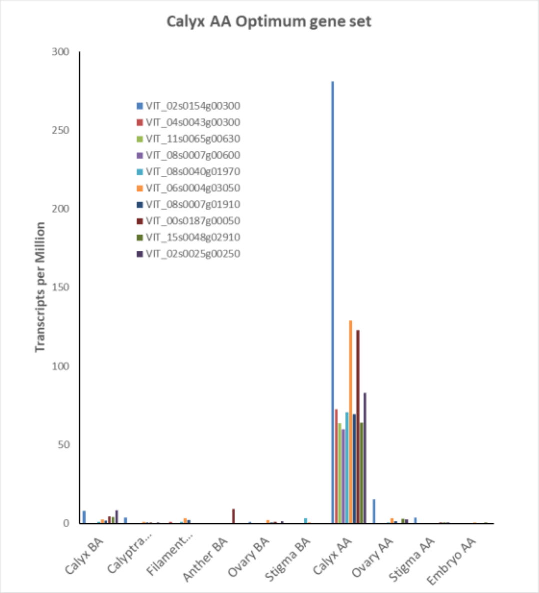

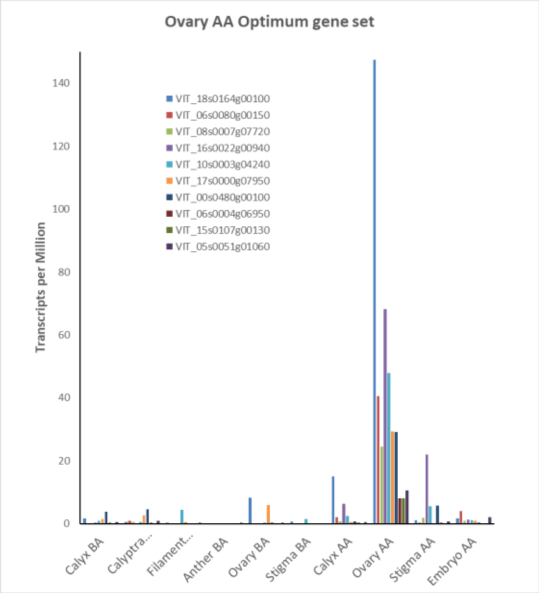

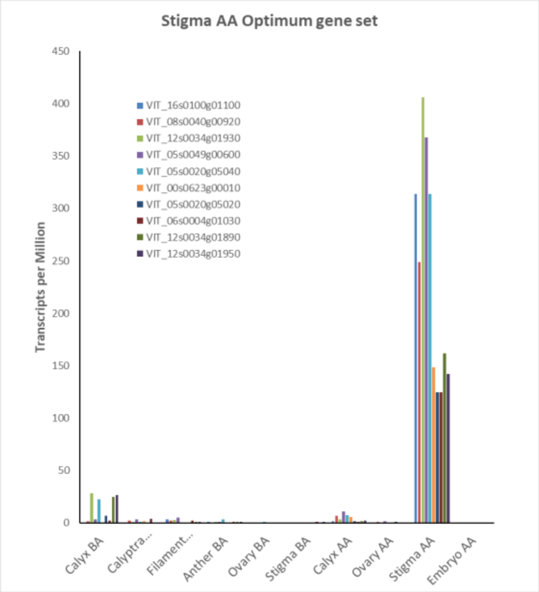

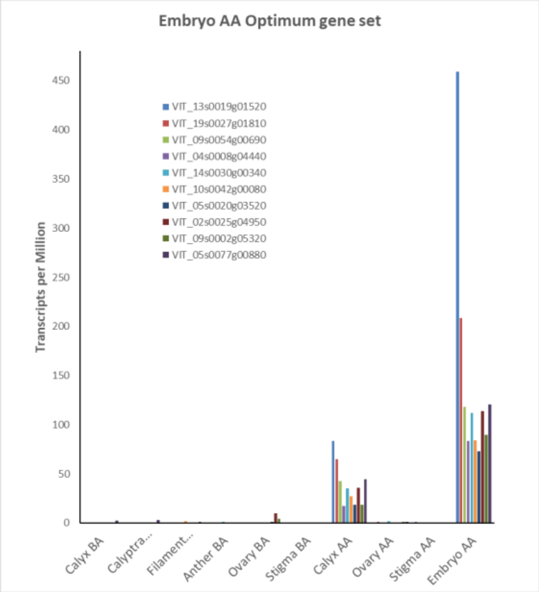

Supplement: Supplementary file 4 — Supplementary Figure 3 [file 41438_2021_635_MOESM4_ESM.pdf]
